# Benchmarking Magnetizabilities with Recent Density Functionals

**DOI:** 10.1021/acs.jctc.0c01190

**Published:** 2021-02-18

**Authors:** Susi Lehtola, Maria Dimitrova, Heike Fliegl, Dage Sundholm

**Affiliations:** † Department of Chemistry, University of Helsinki, P.O. Box 55, A.I. Virtanens plats 1, FI-00014 University of Helsinki, Finland; ‡ Molecular Sciences Software Institute, Blacksburg, Virginia 24061, United States; § Institute of Nanotechnology, KIT, Hermann-von-Helmholtz Platz 1, D-76344 Eggenstein-Leopoldshafen, Germany

## Abstract

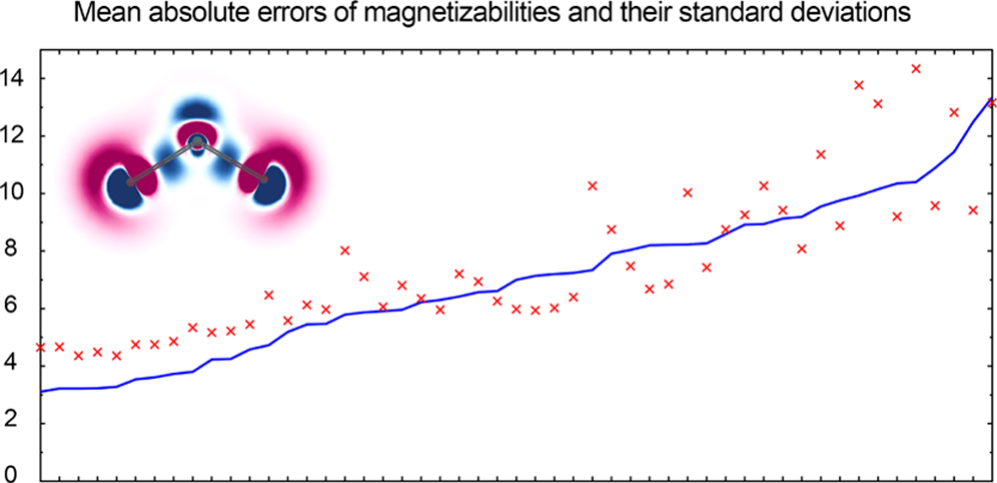

We have assessed the accuracy of the magnetic properties of a set of 51 density functional approximations, including both recently published and already established functionals. The accuracy assessment considers a series of 27 small molecules and is based on comparing the predicted magnetizabilities to literature reference values calculated using coupled-cluster theory with full singles and doubles and perturbative triples [CCSD(T)] employing large basis sets. The most accurate magnetizabilities, defined as the smallest mean absolute error, are obtained with the BHandHLYP functional. Three of the six studied Berkeley functionals and the three range-separated Florida functionals also yield accurate magnetizabilities. Also, some older functionals like CAM-B3LYP, KT1, BHLYP (BHandH), B3LYP, and PBE0 perform rather well. In contrast, unsatisfactory performance is generally obtained with Minnesota functionals, which are therefore not recommended for calculations of magnetically induced current density susceptibilities and related magnetic properties such as magnetizabilities and nuclear magnetic shieldings. We also demonstrate that magnetizabilities can be calculated by numerical integration of magnetizability density; we have implemented this approach as a new feature in the gauge-including magnetically induced current (GIMIC) method. Magnetizabilities can be calculated from magnetically induced current density susceptibilities within this approach even when analytical approaches for magnetizabilities as the second derivative of the energy have not been implemented. The magnetizability density can also be visualized, providing additional information that is not otherwise easily accessible on the spatial origin of magnetizabilities.

## 1 Introduction

Computational methods based on density functional theory (DFT) are commonly used in quantum chemistry because DFT calculations are rather accurate despite their relatively modest computational costs. Older functionals such as the Becke’88–Perdew’86^1,2^ (BP86), Becke’88–Lee–Yang–Parr^1,3^ (BLYP), and Perdew–Burke–Ernzerhof^4,5^ (PBE) functionals at the generalized gradient approximation (GGA) as well as the B3LYP^6^ and PBE0^7,8^ hybrid functionals are still often employed, even though newer functionals with improved accuracy for energies and electronic properties have been developed.

The accuracy and reliability of various density functional approximations (DFAs) have been assessed in a huge number of applications and benchmark studies.^9−17^ It is important to note that functionals that are accurate for energetics may be less suited for calculations of other molecular properties.^16^ In specific, the accuracy of magnetic properties calculated within DFAs has been benchmarked by comparing magnetizabilities and nuclear magnetic shieldings to those obtained from coupled-cluster calculations using large basis sets,^18,19^ although modern DFAs have been less systematically investigated.^16,20−23^ The same also holds for nuclear independent chemical shifts^24−28^ and magnetically induced current density susceptibilities,^29−36^ which have been studied for a large number of molecules, but whose accuracy has never been benchmarked properly.

Magnetizabilities are usually calculated as the second derivative of the electronic energy with respect to the external magnetic perturbation^37−41^
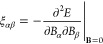
1Such analytic implementations for magnetizabilities exist in several quantum chemistry programs. However, as the magnetic interaction energy can also be written as an integral over the magnetic interaction energy density ρ^**B**^(**r**) given by the scalar product of the magnetically induced current density **J^B^**(**r**) with the vector potential **A^B^**(**r**) of the external magnetic field **B**(30,31,42−45)
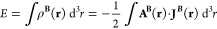
2an approach based on quadrature is also possible. As shown in Section 2, the numerical integration approach for the magnetizability provides additional information about its spatial origin that is not available with the analytic approach based on second derivatives: the tensor components of the magnetizability density defined in Section 2 are scalar functions that can be visualized, and the integration approach can be used to provide detailed information about the origin of the corresponding components of the magnetizability tensor. Similar approaches have been used in the literature for studying spatial contributions to nuclear magnetic shielding constants.^46−53^

We will describe our methods for numerical integration of magnetizabilities using the current density susceptibility in Sections 2 and 3. Then, in Section 4, we will list the studied set of density functionals and present the results in Section 5: the functional benchmark is discussed in Section 5.1 and magnetizability densities and spatial contributions to magnetizabilities are analyzed in Section 5.2. The conclusions of the study are summarized in Section 6. Atomic units are used throughout the text unless stated otherwise, and summation over repeated indices is assumed.

## 2 Theory

The current density **J^B^**(**r**) in eq 2 is formally defined as the real part  of the mechanical momentum density
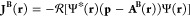
3where **p** = −i∇ is the momentum operator. Substituting eq 2 into eq 1 straightforwardly leads to
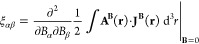
4The current density susceptibility tensor^29−31^ (CDT) is defined as the first derivative of the magnetically induced current density with respect to the components of the external magnetic field in the limit of a vanishing magnetic field^32−35^
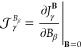
5The vector potential **A**^**B**^(**r**) of an external static homogeneous magnetic field is expressed as
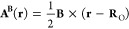
6where **R**_O_ is the chosen gauge origin. The αβ component of the magnetizability tensor can then be obtained from eqs 4–6 as
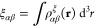
7where the magnetizability density is defined as
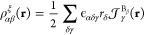
8where ϵ_αδγ_ is the Levi–Civita symbol, α, β, γ, and δ are one of the Cartesian directions (*x*, *y*, *z*), and *r*_δ_ also denotes one of (*x*, *y*, *z*). The components of the magnetizability density tensor ρ_αβ_^ξ^(**r**) are scalar functions that can be visualized to obtain information about the spatial contributions to the corresponding element of the magnetizability tensor ξ_αβ_.

As the isotropic magnetizability (ξ̅) is obtained as the average of the diagonal elements of the magnetizability tensor
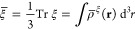
9we introduce the isotropic magnetizability density ρ^ξ̅^(**r**) defined as
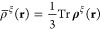
10which yields information about the spatial origin of the isotropic magnetizability, as we demonstrate in Section 5.2.

Although there is freedom with regard to the choice of the gauge origin of **A**^**B**^(**r**), the magnetic flux density **B** is uniquely defined via eq 6, because **B** = ∇ × (**A**(**r**) + ∇*f*(**r**)) holds for any differentiable scalar function *f*(**r**). The exact solution of the Schrödinger equation should also be gauge invariant. However, the use of finite one-particle basis sets introduces gauge dependence in quantum chemical calculations of magnetic properties. The CDT can be made gauge origin independent by using gauge-including atomic orbitals (GIAOs), also known as (a.k.a.) London atomic orbitals (LAOs)^32,54,55^
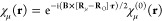
11where i is the imaginary unit and χ_μ_^(0)^(**r**) is a standard atomic-orbital basis function centered at **R**_μ_. GIAOs eliminate the gauge origin from the expression used for calculating the CDT; the expression we use is given in the Supporting Information (SI). Since the expression for the magnetizability density in eqs 7 and 8 can be computed by quadrature, magnetizabilities can be obtained from the CDT even if the corresponding analytical calculation of magnetizabilities as the second derivative of the energy has not been implemented.

## 3 Implementation

The present implementation is based on the gauge-including magnetically induced current (GIMIC) program^56^ and the NUMGRID library,^57^ which are both freely available open-source software. Gauge-independent CDTs can be calculated with GIMIC^32−35^ using the density matrix, magnetically perturbed density matrices, and information about the basis set.

To evaluate eq 7, a molecular integration grid is first generated from atom-centered grids with the NUMGRID library, as described by Becke.^58^ In NUMGRID, the grid weights are scaled according to the Becke partitioning scheme using a Becke hardness of 3;^58^ the atom-centered grids are determined by a radial grid generated as suggested by Lindh et al.,^59^ and angular grids by Lebedev^60^ are used.

Given the quadrature grid, the diagonal elements of the magnetizability tensor are calculated in GIMIC from the Cartesian coordinates of the *n* grid points multiplied with the CDT calculated in the grid points. For example, the ξ_*xx*_ element of the magnetizability tensor is obtained from eq 7 as
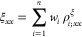
12where the *xx* component of the magnetizability density tensor at grid point *i* with quadrature weight *w_i_* is
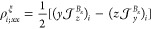
13where  and  are the products of the *z* and *y* components of the CDT calculated in grid point *i* with the Cartesian coordinates *y* and *z* of the grid point, respectively, and the external magnetic field perturbation is along the *x*-axis, *B*_*x*_. The ξ_*yy*_ and ξ_*zz*_ elements are obtained analogously.

## 4 Computational Methods

Calculations are performed for the set of 28 molecules studied in ref (18) that also provides our molecular structures and the CCSD(T) reference values: AlF, C_2_H_4_, C_3_H_4_, CH_2_O, CH_3_F, CH_4_, CO, FCCH, FCN, H_2_C_2_O, H_2_O, H_2_S, H_4_C_2_O, HCN, HCP, HF, HFCO, HOF, LiF, LiH, N_2_, N_2_O, NH_3_, O_3_, OCS, OF_2_, PN, and SO_2_. However, as in ref (18), O_3_ was omitted from the analysis since it is an outlier and due to the fact that the reliability of the CCSD(T) level of theory is not guaranteed for this system: the perturbative triples correction to the magnetizability of O_3_ is −46.2 × 10^–30^ J/T^2^, indicating that the CCSD(T) result might still have large error bars.^18^ The results of this work thus only pertain to the 27 other molecules, as in ref (18).

Electronic structure calculations were performed with Hartree–Fock (HF) and the functionals listed in Tables 1 and 2 using TURBOMOLE 7.5.^110^ Several rungs of Jacob’s ladder were considered when choosing the functionals listed in Tables 1 and 2: local density approximations (LDAs), generalized gradient approximations (GGAs), and meta-GGAs (mGGAs). Several kinds of functionals are also included: (pure) density functional approximations, global hybrid (GH) functionals with a constant amount of HF exchange, and range-separated (RS) hybrids with a given amount of HF exchange in the short range (SR) and the long range (LR). As can be seen in Tables 1 and 2, the evaluated functionals consist of 1 pure LDA, 8 pure GGAs, 8 global hybrid GGAs, 10 range-separated hybrid GGAs, 12 mGGAs, 8 global hybrid mGGAs, and 4 range-separated mGGAs, in addition to HF.

**Table 1:** Functionals at the Local Density Approximation (LDA) and the Generalized Gradient Approximation (GGA) Considered in This Work^f^

functional	hybrid	type	notes	LIBXC ID^a^	references
LDA		LDA		1 + 7	(61−63)
BLYP		GGA		106 + 131	(1, 3), and (64)
BP86		GGA		106 + 132	(1) and (2)
CHACHIYO		GGA		298 + 309	(65) and (66)
KT1		GGA		167	(67)
KT2		GGA		146	(67)
KT3		GGA	PySCF data used	587	(68)
N12		GGA		82 + 80	(69)
PBE		GGA		101 + 130	(4) and (5)
B3LYP	GH	GGA	20% HF	402	(6)
revB3LYP^b^	GH	GGA	20% HF	454	(70)
B97-2	GH	GGA	21% HF	410	(71)
B97-3	GH	GGA	26.9% HF	414	(72)
BHLYP^c^	GH	GGA	50% HF	435	(61, 62), and (73)
BHandHLYP^d^	GH	GGA	50% HF	436	(1) and (73)
PBE0	GH	GGA	25% HF	406	(7) and (8)
QTP-17	GH	GGA	62% HF	416	(74)
N12-SX	RS	GGA	25% SR, 0% LR	81 + 79	(75)
CAM-B3LYP	RS	GGA	19% SR, 65% LR	433	(76)
CAMh-B3LYP^e^	RS	GGA	19% SR, 50% LR		(77)
CAM-QTP-00	RS	GGA	54% SR, 91% LR	490	(78)
CAM-QTP-01	RS	GGA	23% SR, 100% LR	482	(79)
CAM-QTP-02	RS	GGA	28% SR, 100% LR	491	(80)
ωB97	RS	GGA	0% SR, 100% LR	463	(81)
ωB97X	RS	GGA	15.8% SR, 100% LR	464	(81)
ωB97X-D	RS	GGA	22.2% SR, 100% LR	471	(82)
ωB97X-V	RS	GGA	16.7% SR, 100% LR	531	(83)

a Two numbers indicate the exchange and correlation functionals, respectively. A single number indicates an exchange–correlation functional.

b Revised version.

c Following King et al. in refs (84−86), BHLYP is defined as 50% LDA exchange, 50% HF exchange, and 100% LYP correlation. It is sometimes also known as BHandH, which is its keyword in Gaussian.

d BHandHLYP is 50% Becke’88 exchange, 50% HF exchange, and 100% LYP correlation.

e CAMh-B3LYP is defined using the XCFun library with α = 0.19, β = 0.31, and μ = 0.33.

f GH stands for global hybrid and RS for range-separated hybrid. The amount of Hartree–Fock (HF) exchange or exact exchange in the short range (SR) and the long range (LR) is also given.

**Table 2:** Meta-GGA Functionals (mGGA) Considered in This Work^d^

functional	hybrid	type	notes	LIBXC ID^a^	references
B97M-V		mGGA		254	(87)
M06-L		mGGA		449 + 235	(88)
revM06-L^b^		mGGA		293 + 294	(89)
M11-L		mGGA		226 + 75	(90)
MN12-L		mGGA		227 + 74	(91)
MN15-L		mGGA		268 + 269	(92)
TASK		mGGA		707 + 13	(93) and (94)
MVS		mGGA		257 + 83	(95) and (96)
SCAN		mGGA		263 + 267	(97)
rSCAN^c^		mGGA		493 + 494	(98)
TPSS		mGGA		457	(99) and (100)
revTPSS^b^		mGGA		212 + 241	(96) and (101)
TPSSh	GH	mGGA	10% HF	457	(102)
revTPSSh^b^	GH	mGGA	10% HF	458	(96, 101), and (102)
M06	GH	mGGA	27% HF	449 + 235	(103)
revM06^b^	GH	mGGA	40.4% HF	305 + 306	(104)
M06-2X	GH	mGGA	54% HF	450 + 236	(103)
M08-HX	GH	mGGA	52.2% HF	295 + 78	(105)
M08-SO	GH	mGGA	56.8% HF	296 + 77	(105)
MN15	GH	mGGA	44% HF	268 + 269	(106)
M11	RS	mGGA	42.8% SR, 100% LR	297 + 76	(107)
revM11^b^	RS	mGGA	22.5% SR, 100% LR	304 + 172	(108)
MN12-SX	RS	mGGA	25% SR, 0% LR	248 + 73	(75)
ωB97M-V	RS	mGGA	15% SR, 100% LR	531	(109)

a Two numbers indicate the exchange and correlation functionals, respectively. A single number indicates an exchange–correlation functional.

b Revised version.

c Regularized version.

d The notation is the same as in Table 1.

The Dunning aug-cc-pCVQZ basis set^111−115^ (with aug-cc-pVQZ on the hydrogen atoms) and benchmark quality integration grids were employed in all calculations. Universal auxiliary basis sets^116^ were used with the resolution-of-the-identity approximation for the Coulomb interaction in all TURBOMOLE calculations. All density functionals were evaluated in TURBOMOLE with LIBXC,^117^ except the calculations with the recently published CAMh-B3LYP functional for which XCFun was used.^118^ Magnetizabilities were subsequently evaluated with GIMIC by numerical integration of eq 7. The data necessary for evaluating the CDT in GIMIC were obtained from TURBOMOLE calculations of nuclear magnetic resonance (NMR) shielding constants employing GIAOs.^54,55,110,119,120^

Although response calculations are not possible at the moment in the presence of the non-local correlation kernel used in ωB97X-V, B97M-V, and ωB97M-V, we have estimated the importance of the van der Waals (vdW) effects on the magnetic properties by comparing magnetizabilities obtained with orbitals optimized with and without the vdW term in the case of SO_2_. The magnetizability obtained with the vdW-optimized orbitals differed by only 0.4 × 10^–30^ J/T^2^ (0.14%) from that obtained from a calculation where the vdW term was omitted in the orbital optimization. Thus, the vdW term appears to have very little influence on magnetizabilities, as is already well-known in the literature for other properties.^121^ The vdW term was therefore not included in the calculations using the ωB97X-V, B97M-V, and ωB97M-V functionals in this study.

The accuracy of the numerical integration in GIMIC was assessed by comparing the TURBOMOLE/GIMIC magnetizability data to analytical values from PySCF,^122^ in which LIBXC(117) was also used to evaluate the density functionals. Since PySCF does not currently support magnetizability calculations with mGGA functionals or range-separated functionals, further calculations were undertaken with Gaussian 16.^123^ The analytical magnetizabilities from PySCF and Gaussian were found to be in perfect agreement for the studied LDA and GGA functionals available in both codes (LDA, BP86, PBE, PBE0, BLYP, B3LYP, and BHLYP). A comparison of the data from PySCF to the GIMIC data revealed the numerically integrated magnetizabilities to be accurate, as the magnetizabilities agreed within 0.5 × 10^–30^ J/T^2^ for all molecules using the B3LYP, B97-2, B97-3, BLYP, BP86, KT1, KT2, LDA, PBE, and PBE0 functionals; the small discrepancy may arise from the use of the resolution-of-identity approximation^124^ in TURBOMOLE or from the numerical integration of the magnetizability density. A comparison of the raw data for BP86 and B3LYP is given in the SI.

The magnetizabilities calculated with Gaussian and TURBOMOLE using the meta-GGA functionals were found to differ. The discrepancies between the magnetizabilities obtained with the two programs are due to the use of different approaches to handle the gauge invariance of the kinetic energy density in meta-GGAs, which are described in refs (125) and (126) for Gaussian and TURBOMOLE, respectively. We found the TURBOMOLE data to be significantly closer to the CCSD(T) reference values.

Finally, since we found the implementation of the KT3 functional in LIBXC version 5.0.0 used by TURBOMOLE to be flawed, the KT3 results in this study are based on calculations with PySCF with a corrected version of LIBXC.

## 5 Results

### 5.1 Functional Benchmark

**Figure 1:**
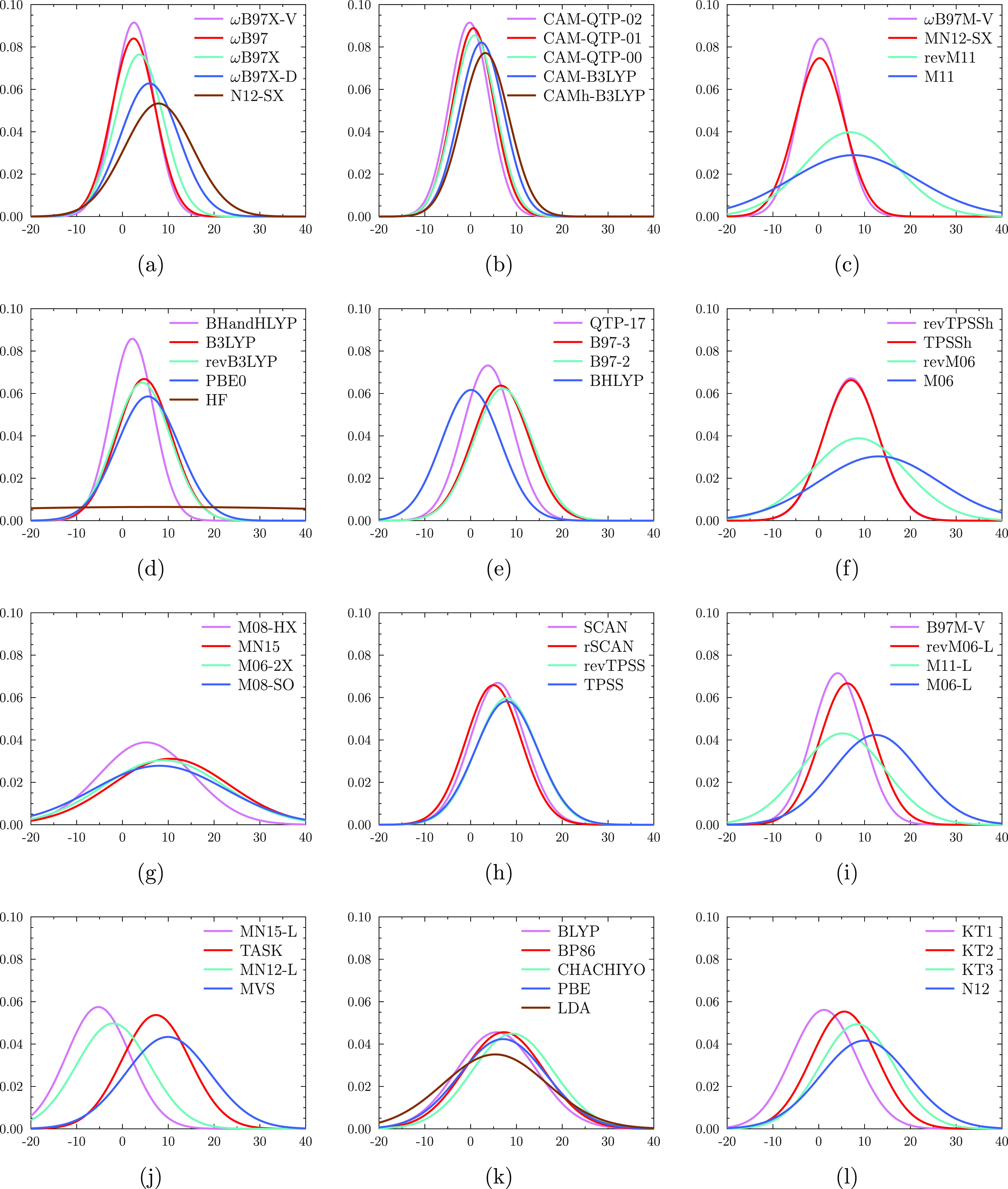
Normal distributions (ND) representing the errors in the magnetizabilities for the 27 benchmark reproduced by the studied functionals, obtained by plotting the data presented in Table 3. The curves are ordered in each figure by increasing standard deviation. The NDs of RS functionals are shown in (a)–(c). The NDs of the GH functionals are shown in (d)–(g). The NDs of the mGGA functionals are shown in (h)–(j). The NDs of the LDA and GGA functionals are shown in (k) and (l).

The deviations of the DFT magnetizabilities from the CCSD(T) reference values of ref (18) are visualized as ideal normal distributions (NDs) in Figure 1. The visualization shows the idealized distribution of the error in the magnetizability for each functional, based on the computed mean errors (ME) and standard deviation of the error (STD) given in Table 3. The raw data on the magnetizabilities and the differences from the CCSD(T) reference are available in the SI. Although the error distributions in Figure 1 are instructive, we will employ mean absolute errors (MAEs) to rank the functionals studied in this work in a simple, unambiguous fashion. The MAEs are also given in Table 3.

**Table 3:** Mean Absolute Errors (MAEs), Mean Errors (MEs), and Standard Deviations (STDs) for the Magnetizabilities of the 27 Studied Molecules in Units of 10^–30^ J/T^2^ from the CCSD(T) Reference with the Studied Functionals^a^

rank	functional	MAE	ME	STD	rank	functional	MAE	ME	STD
1	BHandHLYP	3.11	2.15	4.65	27	revTPSSh	7.14	7.05	5.94
2	CAM-QTP-00	3.22	0.88	4.67	28	TPSSh	7.20	7.07	6.02
3	ωB97X-V	3.22	2.51	4.36	29	B97-2	7.24	7.07	6.40
4	CAM-QTP-01	3.23	0.59	4.49	30	M08-HX	7.34	5.17	10.27
5	CAM-QTP-02	3.28	–0.23	4.36	31	BLYP	7.91	5.69	8.75
6	ωB97	3.54	2.44	4.75	32	N12-SX	8.04	7.89	7.48
7	ωB97M-V	3.61	0.41	4.75	33	revTPSS	8.20	7.86	6.68
8	CAM-B3LYP	3.73	2.38	4.86	34	TPSS	8.22	7.85	6.85
9	MN12-SX	3.80	0.22	5.34	35	revM11	8.23	6.83	10.03
10	CAMh-B3LYP	4.23	3.22	5.17	36	TASK	8.27	7.31	7.43
11	ωB97X	4.25	3.71	5.22	37	BP86	8.59	7.30	8.75
12	QTP-17	4.58	3.77	5.45	38	M11-L	8.92	5.20	9.26
13	BHLYP	4.73	0.10	6.47	39	revM06	8.94	8.67	10.27
14	B97M-V	5.19	4.13	5.58	40	PBE	9.13	7.07	9.42
15	revB3LYP	5.45	4.34	6.13	41	KT3	9.19	8.38	8.08
16	B3LYP	5.47	4.72	5.97	42	LDA	9.55	5.37	11.36
17	MN12-L	5.79	–2.03	8.02	43	CHACHIYO	9.76	9.17	8.88
18	KT1	5.87	1.15	7.11	44	M11	9.93	7.61	13.77
19	rSCAN	5.91	5.00	6.06	45	M06-2X	10.15	9.01	13.12
20	PBE0	5.96	5.56	6.81	46	MVS	10.35	9.92	9.20
21	ωB97X-D	6.22	5.89	6.35	47	M08-SO	10.40	8.09	14.34
22	SCAN	6.30	5.89	5.96	48	N12	10.89	10.01	9.58
23	KT2	6.42	5.58	7.21	49	MN15	11.45	10.45	12.82
24	MN15-L	6.57	–5.27	6.94	50	M06-L	12.49	12.45	9.42
25	B97-3	6.61	6.61	6.26	51	M06	13.34	13.11	13.16
26	revM06-L	7.00	6.23	5.98	52	HF	18.40	7.48	61.81

a The functionals are ordered in increasing MAE.

Examination of the data in Table 3 shows that range-separated (RS) functionals generally yield accurate magnetizabilities. Judged by the mean absolute error, the best performance is obtained with the BHandHLYP GH functional. BHandHLYP is followed by 10 RS functionals, which have much sharper distributions than the rest of the studied functionals. The best performing RS functionals are three of the six Berkeley RS functionals (ωB97X-V, ωB97, and ωB97M-V) and the three RS functionals from the University of Florida’s Quantum Theory Project (QTP) CAM-QTP-00, CAM-QTP-01, and CAM-QTP-02. Five of these functionals have 100% long-range (LR) HF exchange, while the CAM-QTP-00 functional has 91% LR HF exchange. The two other RS Berkeley functionals with 100% LR exchange are ranked 11th (ωB97X) and 21st (ωB97X-D) among the studied functionals. The NDs of the studied RS GGA functionals are shown in Figure 1a,b, whereas the NDs of the studied RS mGGA functionals are shown in Figure 1c.

The CAM-B3LYP (65% LR HF exchange) and CAMh-B3LYP (50% LR HF exchange) functionals are among the top 10 functionals (ranked 8th and 10th, respectively). CAM-B3LYP was designed for the accurate description of charge transfer excitations in a dipeptide model,^76^ while CAMh-B3LYP functional is aimed at excitation energies of biochromophores.^77^

The best Minnesota functional, MN12-SX, is ranked 9th. MN12-SX is a highly parameterized functional with 58 parameters that is known to require the use of extremely accurate integration grids.^13^ Furthermore, since MN12-SX is an RS functional with HF exchange only in the short range (SR), it may have problems modeling magnetic properties of antiaromatic molecules sustaining strong ring currents in the paratropic (nonclassical) direction.^127−129^ We illustrate this with calculations on the strongly antiaromatic tetraoxa isophlorin molecule in the Supporting Information: MN12-SX yields a magnetizability that is 4 times larger than the local second-order Møller–Plesset perturbation theory (LMP2) reference value, while the magnetizabilities from BHandHLYP and CAM-B3LYP are in good agreement with LMP2. The N12-SX functional ranked 32nd is also an RS functional with 0% LR exchange. The RS Minnesota functionals with 100% LR HF exchange (M11 and revM11) have large MAEs of 9.93 × 10^–30^ J/T^2^ and 8.87 × 10^–30^ J/T^2^ and are ranked 44th and 35th, respectively.

The best global hybrid (GH) functional is BHandHLYP, which is ranked first among all functionals of this study, as was already mentioned above. Among GHs, BHandHLYP is followed by QTP-17, which is ranked 12th. Old and established GH functionals like BHLYP a.k.a. BHandH, B3LYP, and PBE0 perform almost as well as QTP-17 and are ranked 13th, 16th, and 20th, respectively. The performance of revB3LYP is practically the same as that of B3LYP; the same holds for revTPSSh and TPSSh. The other established GH functionals like B97-2, B97-3, and TPSSh and newer ones like revTPSSh and M08-HX are found in the beginning of the second half of the ranking list, whereas M08-SO, M06, revM06, M06-2X, MN15, and M06 are ranked between 39th and 51st. The NDs of the GH functionals are compared in Figure 1d–g.

B97M-V, at the 14th place, is the best pure mGGA functional. The rSCAN and SCAN functionals are ranked 19th and 22th, respectively, whereas revTPSS and TPSS appear at positions 33 and 34, respectively. The pure mGGA functionals of the Minnesota series are ranked 17th (MN12-L), 24th (MN15-L), 26th (revM06-L), and 50th (M06-L). The performance of the Minnesota pure mGGA functionals, excluding M06-L, is about the same as that of TASK and the other mGGA functionals. The magnetizabilities calculated with the revised M06-L (revM06-L) functional are more accurate than those with M06-L. The MVS mGGA functional is ranked 46th. The NDs for the mGGA functionals are shown in Figure 1h–j.

The magnetizabilities calculated with several of the Minnesota functionals are inaccurate. Seven of the eight worst performing functionals (M11, M06-2X, MVS, M08-SO, N12, MN15, M06-L, and M06) in Table 3 are Minnesota functionals. Five other Minnesota functionals are also ranked in the lower half, placing 30th (M08-HX), 32th (N12-SX), 35th (revM11), 38th (M11-L), and 39th (revM06).

The KT1 and KT2 functionals are the best GGA functionals, ranking 18th and 23rd, respectively; both KT1 and KT2 have been optimized for NMR shieldings.^67^ The older commonly used GGAs i.e., BLYP, BP86, and PBE are ranked 31st, 37th, and 40th, respectively, which is only slightly better than KT3 ranked 41st and LDA ranked 42nd. The CHACHIYO and N12 functionals, which are newer GGAs, are ranked 43rd and 48th, respectively. The NDs of the GGA functionals and the LDA are shown in Figure 1k,l.

The magnetizabilities calculated at the HF level are significantly less accurate and have a much larger MAE-STD than those obtained at the DFT levels, and we cannot recommend the use of HF for magnetic properties.

### 5.2 Magnetizability Densities

**Figure 2:**
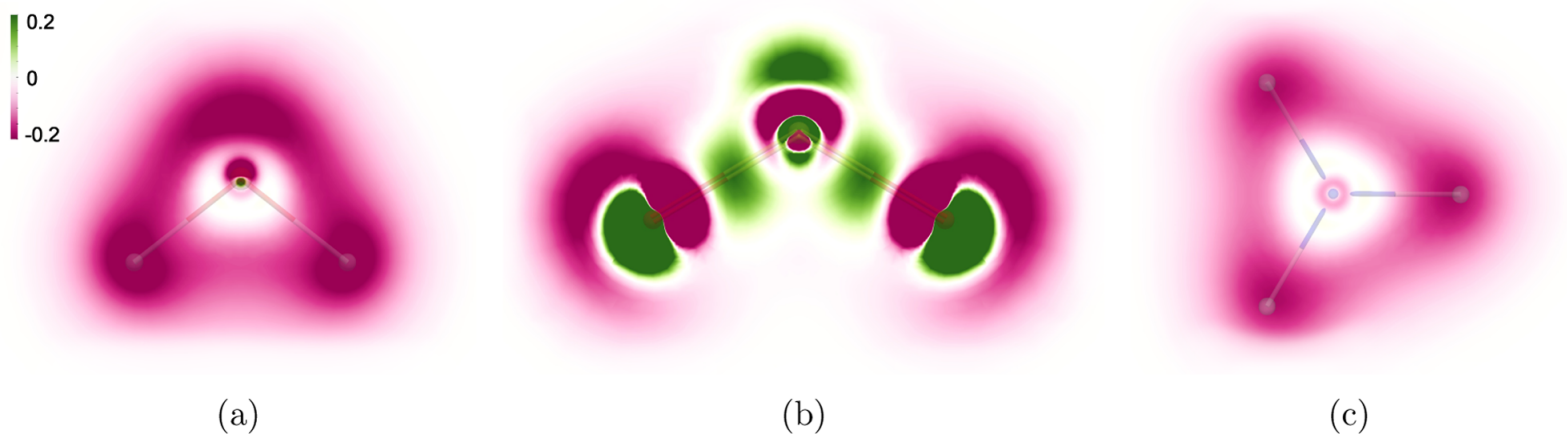
Visualization of the isotropic magnetizability density ρ̅^ξ^(**r**) (eq 10) shown in the molecular plane of H_2_O (a) and SO_2_ (b) as well as in the plane formed by the hydrogen atoms of NH_3_ (c), positioned 0.06 *a*_0_ away from the N atom toward the hydrogen atoms. Negative contributions are shown in pink and positive ones in green. The gauge origin **R**_O_ is (0, 0, 0) *a*_0_.

**Figure 3:**
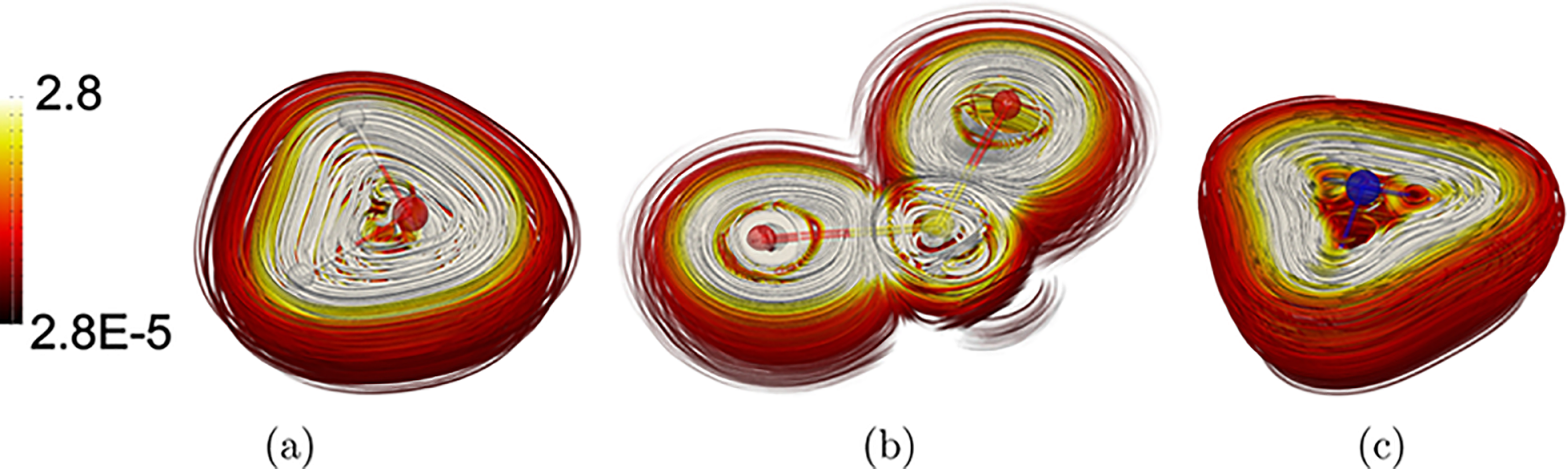
Streamline representation of the CDT (eq 5) of H_2_O (a), SO_2_ (b), and NH_3_ (c). The CDT is calculated with the magnetic field perpendicular to the molecular plane of H_2_O and SO_2_ as well as with it along the symmetry axis of NH_3_. The color scale represents the strength of the CDT in nAT^–1^*a*_0_^–2^.

Spatial contributions to the magnetizability densities, i.e., the integrand in eq 7, are illustrated for H_2_O, NH_3_, and SO_2_ in Figure 2, with Figure 3 showing the corresponding CDTs. The magnetizability densities are calculated with the gauge origin of the external magnetic field (**R**_O_) at (*x*, *y*, *z*) = (0, 0, 0). In the calculations on H_2_O and SO_2_, the magnetic field perturbation is perpendicular to the molecular plane, while for NH_3_, the perturbation is parallel to the *C*_3_ symmetry axis. In the case of H_2_O, the current density flux around the whole molecule (Figure 3) leads to the ring-shaped contribution shown in Figure 2. The magnetic field along the symmetry axis of NH_3_ also results in a current density flux around the molecule at the hydrogen atoms (Figure 3), giving rise to a similar ring-shaped contribution shown in Figure 2.

The isotropic magnetizability density of SO_2_ shown in Figure 2 has positive (green) and negative (pink) values. Calculations of the CDT show that the oxygen atoms sustain a strong diatropic atomic CDT that flows around the atom, whereas the atomic CDT of the sulfur atom is much weaker (Figure 3). The p-orbital shaped contributions to the magnetizability density of SO_2_ around the oxygen atoms in Figure 2 originate from the atomic CDTs. The patterns of the CDT of H_2_O and SO_2_ lead to the different magnetizability densities shown in Figure 2a,b, respectively. The positive magnetizability densities in H_2_O and NH_3_ are extremely localized close to the atomic nuclei, also because of the vortices of the atomic CDT.

The magnetizability density depends on the gauge origin of the vector potential of the external magnetic field, even though the magnetizability is independent of the gauge origin.^43^ The magnetizability densities for H_2_O, NH_3_, and SO_2_ calculated with the gauge origin at **R**_O_ = (1, 1, 1) *a*_0_ are shown in the SI. The contribution of the choice of the gauge origin to the magnetizability computed from eq 7 vanishes when the CDT fulfills the charge conservation condition^29^
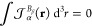
14Calculating the magnetizability for NH_3_ with a gauge origin set to **R**_O_ = (100, 100, 100) *a*_0_ yielded a value that differs by 0.32% from the one computed for **R**_O_ = (0, 0, 0). When the gauge origin is set to **R**_O_ = (1, 1, 1) *a*_0_, the deviation is 2 orders of magnitude smaller because the change in the magnetizability depends linearly on the relative position of the gauge origin. The magnetizabilities of H_2_O and SO_2_ also change by only 0.46 and 0.03% when moving the gauge origin from (0, 0, 0) *a*_0_ to (100, 100, 100) *a*_0_, respectively, showing that charge conservation is practically fulfilled in our calculations. All other positions than (0, 0, 0) for the gauge origin lead to a small, spurious CDT contribution to the magnetizability density.

The GIAO ansatz modifies the atomic orbitals leading to a magnetic response of an external magnetic field that is correct to the first order for the one-center problem.^30,130^ Even though GIAOs do not guarantee that the integral condition for the charge conservation of the CDT is fulfilled,^131^ the basis set convergence is faster and the leakage of the CDT is much smaller when GIAOs are used.^32^

## 6 Conclusions

**Figure 4:**
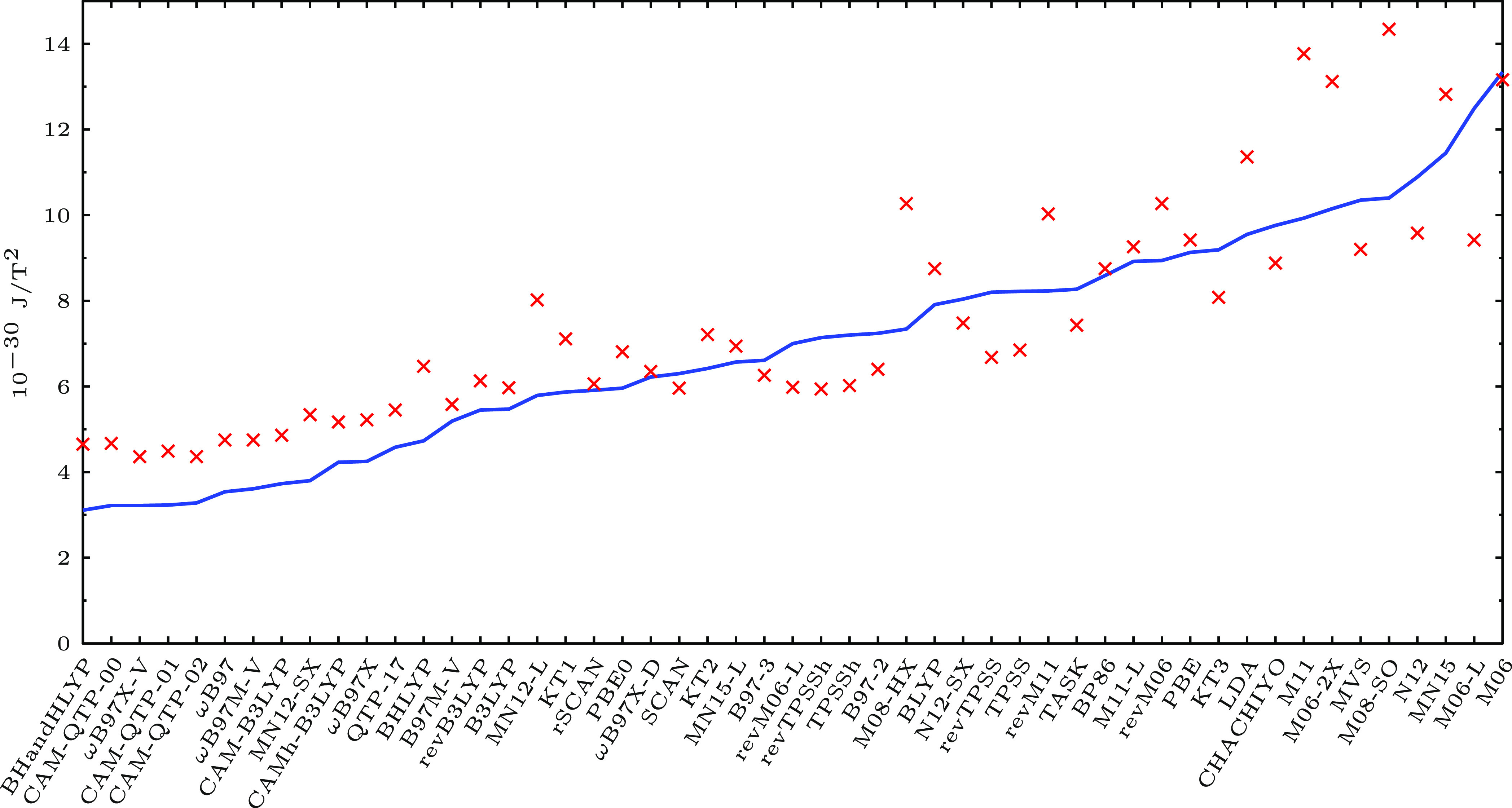
Mean absolute errors (the blue solid line) as well as the errors’ standard deviations (red crosses) of the magnetizabilities in 10^–30^ J/T^2^ of the 27 studied molecules obtained with the 51 functionals compared to the CCSD(T) reference.

We have calculated magnetizabilities for a series of small molecules using both recently published density functionals, as well as older, established density functionals. The accuracy of the magnetizabilities predicted by the various density functional approximations has been assessed by comparison to coupled-cluster calculations with singles and doubles and perturbative triples [CCSD(T)] reported by Lutnæs et al.^18^ Our results are summarized graphically in Figure 4: the top functionals afford both small mean absolute errors and standard deviations, but the same is not true for all recently suggested functionals.

Numerical methods for calculating magnetizabilities based on the quadrature of the magnetizability density have been implemented. We have shown that this method allows studies of spatial contributions to the magnetizabilities by visualization of the magnetizability density. The method has been employed to calculate magnetizabilities from magnetically induced current density susceptibilities, which were obtained from TURBOMOLE calculations of nuclear magnetic shielding constants. Thus, magnetizabilities can be calculated in this way with TURBOMOLE even though analytical methods to calculate magnetizabilities as the second derivative of the energy are not yet available in this program. Further information about spatial contributions to the magnetizability could be obtained in the present approach by studying atomic contributions and investigating the positive and negative parts of the integrands separately in analogy to our recent work on nuclear magnetic shieldings in ref (53), which may be studied in the future work.

Our calculations show that the most accurate magnetizabilities (judged by the smallest MAE) for the studied database are obtained with BHandHLYP, which is an old global hybrid with 50% HF exchange and 50% B88 exchange. The calculations also show that the modern range-separated functionals with 100% long-range HF exchange developed by Head-Gordon and co-workers and by Bartlett and co-workers yield accurate magnetizabilities for the database. Calculations with other range-separated functionals like CAM-B3LYP and CAMh-B3LYP as well as with global hybrid functionals like QTP-17, BHLYP a.k.a. BHandH, B3LYP, and PBE0 yield relatively accurate magnetizabilities for the studied molecules. Meta-GGA functionals are found to yield somewhat better magnetizabilities than GGA and LDA functionals.

However, functionals developed by Truhlar and co-workers do not appear to be well-aimed for calculations of magnetizabilities and other magnetic properties that involve magnetically induced current densities. Magnetizabilities calculated using the popular M06-2X functional are found to be unreliable, and we do not recommend the use of the M06-2X functional in calculations of nuclear magnetic shieldings, magnetizabilities, ring-current strengths, and other magnetic properties that depend on magnetically induced current density susceptibilities. Previous studies have also suggested that the M06-2X functional sometimes underestimates magnetizabilities and ring-current strengths.^128,129,132^ Revised versions of Minnesota functionals have been studied in this work and found to yield somewhat more accurate magnetizabilities than the original parameterizations. However, the revised versions also still appear on the second half of the ranking list.

## Supporting Information Available

Calculated magnetizabilities for the studied molecules and functionals as well as the difference between the calculated magnetizabilities and the CCSD(T) reference values taken from ref (18); magnetizability densities calculated with the gauge origin **R**_O_ = (1, 1, 1) *a*_0_; and additional calculations on tetraoxa isophlorin are discussed (PDF)

## References

[ref1] Becke A. D. Density-functional exchange-energy approximation with correct asymptotic behavior. Phys. Rev. A 1988, 38, 3098 10.1103/PhysRevA.38.3098. 9900728

[ref2] Perdew J. P. Density-functional approximation for the correlation energy of the inhomogeneous electron gas. Phys. Rev. B 1986, 33, 8822 10.1103/PhysRevB.33.8822. 9938299

[ref3] Lee C.; Yang W.; Parr R. G. Development of the Colle-Salvetti correlation-energy formula into a functional of the electron density. Phys. Rev. B 1988, 37, 785 10.1103/PhysRevB.37.785. 9944570

[ref4] Perdew J. P.; Burke K.; Ernzerhof M. Generalized Gradient Approximation Made Simple. Phys. Rev. Lett. 1996, 77, 3865 10.1103/PhysRevLett.77.3865. 10062328

[ref5] Perdew J. P.; Burke K.; Ernzerhof M. Generalized Gradient Approximation Made Simple [Phys. Rev. Lett. 77, 3865 (1996)]. Phys. Rev. Lett. 1997, 78, 1396 10.1103/PhysRevLett.78.1396. 10062328

[ref6] Stephens P. J.; Devlin F. J.; Chabalowski C. F.; Frisch M. J. Ab Initio Calculation of Vibrational Absorption and Circular Dichroism Spectra Using Density Functional Force Fields. J. Phys. Chem. A 1994, 98, 11623 10.1021/j100096a001.

[ref7] Adamo C.; Barone V. Toward reliable density functional methods without adjustable parameters: The PBE0 model. J. Chem. Phys. 1999, 110, 6158 10.1063/1.478522.

[ref8] Ernzerhof M.; Scuseria G. E. Assessment of the Perdew-Burke-Ernzerhof exchange-correlation functional. J. Chem. Phys. 1999, 110, 5029 10.1063/1.478401. 15268348

[ref9] Silva-Junior M. R.; Schreiber M.; Sauer S. P. A.; Thiel W. Benchmarks for electronically excited states: Time-dependent density functional theory and density functional theory based multireference configuration interaction. J. Chem. Phys. 2008, 129, 104103 10.1063/1.2973541. 19044904

[ref10] Sauer S. P. A.; Schreiber M.; Silva-Junior M. R.; Thiel W. Benchmarks for Electronically Excited States: A Comparison of Noniterative and Iterative Triples Corrections in Linear Response Coupled Cluster Methods: CCSDR(3) versus CC3. J. Chem. Theory Comput. 2009, 5, 555–564. 10.1021/ct800256j. 26610222

[ref11] Silva-Junior M. R.; Schreiber M.; Sauer S. P. A.; Thiel W. Benchmarks of electronically excited states: Basis set effects on CASPT2 results. J. Chem. Phys. 2010, 133, 174318 10.1063/1.3499598. 21054043

[ref12] Laurent A. D.; Jacquemin D. TD-DFT benchmarks: A review. Int. J. Quantum Chem. 2013, 113, 2019–2039. 10.1002/qua.24438.

[ref13] Mardirossian N.; Head-Gordon M. How Accurate Are the Minnesota Density Functionals for Noncovalent Interactions, Isomerization Energies, Thermochemistry, and Barrier Heights Involving Molecules Composed of Main-Group Elements?. J. Chem. Theory Comput. 2016, 12, 4303–4325. 10.1021/acs.jctc.6b00637. 27537680

[ref14] Goerigk L.; Grimme S. A thorough benchmark of density functional methods for general main group thermochemistry, kinetics, and noncovalent interactions. Phys. Chem. Chem. Phys. 2011, 13, 6670 10.1039/c0cp02984j. 21384027

[ref15] Mardirossian N.; Head-Gordon M. Thirty years of density functional theory in computational chemistry: an overview and extensive assessment of 200 density functionals. Mol. Phys. 2017, 115, 2315–2372. 10.1080/00268976.2017.1333644.

[ref16] Stoychev G. L.; Auer A. A.; Izsák R.; Neese F. Self-Consistent Field Calculation of Nuclear Magnetic Resonance Chemical Shielding Constants Using Gauge-Including Atomic Orbitals and Approximate Two-Electron Integrals. J. Chem. Theory Comput. 2018, 14, 619–637. 10.1021/acs.jctc.7b01006. 29301077

[ref17] Grabarek D.; Andruniów T. Assessment of Functionals for TDDFT Calculations of One- and Two-Photon Absorption Properties of Neutral and Anionic Fluorescent Proteins Chromophores. J. Chem. Theory Comput. 2019, 15, 490–508. 10.1021/acs.jctc.8b00769. 30485096

[ref18] Lutnæs O. B.; Teale A. M.; Helgaker T.; Tozer D. J.; Ruud K.; Gauss J. Benchmarking density-functional-theory calculations of rotational g tensors and magnetizabilities using accurate coupled-cluster calculations. J. Chem. Phys. 2009, 131, 144104 10.1063/1.3242081. 19831430

[ref19] Teale A. M.; Lutnæs O. B.; Helgaker T.; Tozer D. J.; Gauss J. Benchmarking density-functional theory calculations of NMR shielding constants and spin-rotation constants using accurate coupled-cluster calculations. J. Chem. Phys. 2013, 138, 024111 10.1063/1.4773016. 23320672

[ref20] Zhao Y.; Truhlar D. G. Improved Description of Nuclear Magnetic Resonance Chemical Shielding Constants Using the M06-L Meta-Generalized-Gradient-Approximation Density Functional. J. Phys. Chem. A 2008, 112, 6794–6799. 10.1021/jp804583d. 18613657

[ref21] Johansson M. P.; Swart M. Magnetizabilities at Self-Interaction-Corrected Density Functional Theory Level. J. Chem. Theory Comput. 2010, 6, 3302–3311. 10.1021/ct100235b. 26617084

[ref22] Gromov O. I.; Kuzin S. V.; Golubeva E. N. Performance of DFT methods in the calculation of isotropic and dipolar contributions to ^14^N hyperfine coupling constants of nitroxide radicals. J. Mol. Model. 2019, 25, 93 10.1007/s00894-019-3966-y. 30859325

[ref23] Zuniga-Gutierrez B.; Geudtner G.; Köster A. M. Magnetizability tensors from auxiliary density functional theory. J. Chem. Phys. 2012, 137, 094113 10.1063/1.4749243. 22957561

[ref24] Chen Z.; Wannere C. S.; Corminboeuf C.; Puchta R.; Schleyer P. v. R. Nucleus-independent chemical shifts (NICS) as an aromaticity criterion. Chem. Rev. 2005, 105, 3842–3888. 10.1021/cr030088+. 16218569

[ref25] Solà M.; Feixas F.; Jiménez-Halla J. O. C.; Matito E.; Poater J. A Critical Assessment of the Performance of Magnetic and Electronic Indices of Aromaticity. Symmetry 2010, 2, 1156–1179. 10.3390/sym2021156.

[ref26] Rosenberg M.; Dahlstrand C.; Kilså K.; Ottosson H. Excited State Aromaticity and Antiaromaticity: Opportunities for Photophysical and Photochemical Rationalizations. Chem. Rev. 2014, 114, 5379–5425. 10.1021/cr300471v. 24712859

[ref27] Gershoni-Poranne R.; Stanger A. Magnetic criteria of aromaticity. Chem. Soc. Rev. 2015, 44, 6597–6615. 10.1039/C5CS00114E. 26035305

[ref28] Gajda Ł.; Kupka T.; Broda M. A.; Leszczyńska M.; Ejsmont K. Method and basis set dependence of the NICS indexes of aromaticity for benzene. Magn. Reson. Chem. 2018, 56, 265–275. 10.1002/mrc.4690. 29211311

[ref29] Sambe H. Properties of induced electron current density of a molecule under a static uniform magnetic field. J. Chem. Phys. 1973, 59, 555 10.1063/1.1679845.

[ref30] Lazzeretti P. Ring currents. Prog. Nucl. Magn. Reson. Spectrosc. 2000, 36, 1–88. 10.1016/S0079-6565(99)00021-7.

[ref31] Lazzeretti P. Current density tensors. J. Chem. Phys. 2018, 148, 134109 10.1063/1.5025046. 29626873

[ref32] Jusélius J.; Sundholm D.; Gauss J. Calculation of current densities using gauge-including atomic orbitals. J. Chem. Phys. 2004, 121, 3952–3963. 10.1063/1.1773136. 15332941

[ref33] Taubert S.; Sundholm D.; Jusélius J. Calculation of spin-current densities using gauge-including atomic orbitals. J. Chem. Phys. 2011, 134, 054123 10.1063/1.3549567. 21303108

[ref34] Fliegl H.; Taubert S.; Lehtonen O.; Sundholm D. The gauge including magnetically induced current method. Phys. Chem. Chem. Phys. 2011, 13, 20500 10.1039/c1cp21812c. 21909556

[ref35] Sundholm D.; Fliegl H.; Berger R. J. Calculations of magnetically induced current densities: theory and applications. Wiley Interdiscip. Rev.: Comput. Mol. Sci. 2016, 6, 639–678. 10.1002/wcms.1270.

[ref36] Fliegl H.; Valiev R.; Pichierri F.; Sundholm D. Theoretical studies as a tool for understanding the aromatic character of porphyrinoid compounds. Chem. Modell. 2018, 1–42. 10.1039/9781788010719-00001.

[ref37] Ruud K.; Helgaker T.; Bak K. L.; Jørgensen P.; Jensen H. J. A. Hartree-Fock limit magnetizabilities from London orbitals. J. Chem. Phys. 1993, 99, 3847 10.1063/1.466131.

[ref38] Ruud K.; Skaane H.; Helgaker T.; Bak K. L.; Jørgensen P. Magnetizability of Hydrocarbons. J. Am. Chem. Soc. 1994, 116, 10135–10140. 10.1021/ja00101a036.

[ref39] Ruud K.; Helgaker T.; Bak K. L.; Jørgensen P.; Olsen J. Accurate magnetizabilities of the isoelectronic series BeH^–^, BH, and CH^+^. The MCSCF-GIAO approach. Chem. Phys. 1995, 195, 157–169. 10.1016/0301-0104(95)00052-P.

[ref40] Loibl S.; Schütz M. Magnetizability and rotational g tensors for density fitted local second-order Møller-Plesset perturbation theory using gauge-including atomic orbitals. J. Chem. Phys. 2014, 141, 024108 10.1063/1.4884959. 25028000

[ref41] Helgaker T.; Coriani S.; Jørgensen P.; Kristensen K.; Olsen J.; Ruud K. Recent Advances in Wave Function-Based Methods of Molecular-Property Calculations. Chem. Rev. 2012, 112, 543–631. 10.1021/cr2002239. 22236047

[ref42] Jameson C. J.; Buckingham A. D. Nuclear magnetic shielding density. J. Phys. Chem. B 1979, 83, 3366–3371. 10.1021/j100489a011.

[ref43] Jameson C. J.; Buckingham A. D. Molecular electronic property density functions: The nuclear magnetic shielding density. J. Chem. Phys. 1980, 73, 5684–5692. 10.1063/1.440045.

[ref44] Fowler P. W.; Steiner E.; Cadioli B.; Zanasi R. Distributed-gauge calculations of current density maps, magnetizabilities, and shieldings for a series of neutral and dianionic fused tetracycles: pyracylene (C_14_H_8_), acepleiadylene (C_16_H_10_), and dipleiadiene (C_18_H_12_). J. Phys. Chem. A 1998, 102, 7297–7302. 10.1021/jp981231j.

[ref45] Iliaš M.; Jensen H. J. A.; Bast R.; Saue T. Gauge origin independent calculations of molecular magnetisabilities in relativistic four-component theory. Mol. Phys. 2013, 111, 1373–1381. 10.1080/00268976.2013.798436.

[ref46] Steiner E.; Fowler P. W. On the orbital analysis of magnetic properties. Phys. Chem. Chem. Phys. 2004, 6, 261–272. 10.1039/B312289C.

[ref47] Pelloni S.; Ligabue A.; Lazzeretti P. Ring-current models from the differential Biot-Savart law. Org. Lett. 2004, 6, 4451–4454. 10.1021/ol048332m. 15548048

[ref48] Ferraro M. B.; Lazzeretti P.; Viglione R. G.; Zanasi R. Understanding proton magnetic shielding in the benzene molecule. Chem. Phys. Lett. 2004, 390, 268–271. 10.1016/j.cplett.2004.04.022.

[ref49] Soncini A.; Fowler P.; Lazzeretti P.; Zanasi R. Ring-current signatures in shielding-density maps. Chem. Phys. Lett. 2005, 401, 164–169. 10.1016/j.cplett.2004.11.044.

[ref50] Ferraro M. B.; Faglioni F.; Ligabue A.; Pelloni S.; Lazzeretti P. Ring current effects on nuclear magnetic shielding of carbon in the benzene molecule. Magn. Reson. Chem. 2005, 43, 316–320. 10.1002/mrc.1536. 15625723

[ref51] Acke G.; Van Damme S.; Havenith R. W. A.; Bultinck P. Interpreting the behavior of the NICSzz by resolving in orbitals, sign, and positions. J. Comput. Chem. 2018, 39, 511–519. 10.1002/jcc.25095. 29098697

[ref52] Acke G.; Van Damme S.; Havenith R. W. A.; Bultinck P. Quantifying the conceptual problems associated with the isotropic NICS through analyses of its underlying density. Phys. Chem. Chem. Phys. 2019, 21, 3145–3153. 10.1039/C8CP07343K. 30675885

[ref53] Jinger R. K.; Fliegl H.; Bast R.; Dimitrova M.; Lehtola S.; Sundholm D. Spatial Contributions to Nuclear Magnetic Shieldings. J. Phys. Chem. A 2021, 10.1021/acs.jpca.0c10884. PMC8023705 33605721

[ref54] Ditchfield R. Self-consistent perturbation theory of diamagnetism. I. A gauge-invariant LCAO method for N.M.R. chemical shifts. Mol. Phys. 1974, 27, 789–807. 10.1080/00268977400100711.

[ref55] Wolinski K.; Hinton J. F.; Pulay P. Efficient implementation of the gauge-independent atomic orbital method for NMR chemical shift calculations. J. Am. Chem. Soc. 1990, 112, 8251–8260. 10.1021/ja00179a005.

[ref56] GIMIC, version 2.0, a current density program. Can be freely downloaded from https://github.com/qmcurrents/gimic.

[ref57] Bast R. NUMGRID: Numerical Integration Grid for Molecules, 2020. https://doi.org/10.5281/zenodo.1470276.

[ref58] Becke A. D. A multicenter numerical integration scheme for polyatomic molecules. J. Chem. Phys. 1988, 88, 2547–2553. 10.1063/1.454033.

[ref59] Lindh R.; Malmqvist P.-Å.; Gagliardi L. Molecular integrals by numerical quadrature. I. Radial integration. Theor. Chem. Acc. 2001, 106, 178–187. 10.1007/s002140100263.

[ref60] Lebedev V. I. A quadrature formula for the sphere of 59th algebraic order of accuracy. Russ. Acad. Sci. Dokl. Math. 1995, 50, 283–286.

[ref61] Bloch F. Bemerkung zur Elektronentheorie des Ferromagnetismus und der elektrischen Leitfähigkeit. Z. Phys. 1929, 57, 545 10.1007/BF01340281.

[ref62] Dirac P. A. M. Note on Exchange Phenomena in the Thomas Atom. Math. Proc. Cambridge Philos. Soc. 1930, 26, 376 10.1017/S0305004100016108.

[ref63] Vosko S. H.; Wilk L.; Nusair M. Accurate spin-dependent electron liquid correlation energies for local spin density calculations: a critical analysis. Can. J. Phys. 1980, 58, 1200 10.1139/p80-159.

[ref64] Miehlich B.; Savin A.; Stoll H.; Preuss H. Results obtained with the correlation energy density functionals of becke and Lee, Yang and Parr. Chem. Phys. Lett. 1989, 157, 200 10.1016/0009-2614(89)87234-3.

[ref65] Chachiyo T.; Chachiyo H. Simple and Accurate Exchange Energy for Density Functional Theory. Molecules 2020, 25, 3485 10.3390/molecules25153485. PMC7436057 32751903

[ref66] Chachiyo T.; Chachiyo H. Understanding electron correlation energy through density functional theory. Comput. Theor. Chem. 2020, 1172, 112669 10.1016/j.comptc.2019.112669.

[ref67] Keal T. W.; Tozer D. J. The exchange-correlation potential in Kohn-Sham nuclear magnetic resonance shielding calculations. J. Chem. Phys. 2003, 119, 3015 10.1063/1.1590634.

[ref68] Keal T. W.; Tozer D. J. A semiempirical generalized gradient approximation exchange-correlation functional. J. Chem. Phys. 2004, 121, 5654–5660. 10.1063/1.1784777. 15366989

[ref69] Peverati R.; Truhlar D. G. Exchange-Correlation Functional with Good Accuracy for Both Structural and Energetic Properties while Depending Only on the Density and Its Gradient. J. Chem. Theory Comput. 2012, 8, 2310 10.1021/ct3002656. 26588964

[ref70] Lu L.; Hu H.; Hou H.; Wang B. An improved B3LYP method in the calculation of organic thermochemistry and reactivity. Comput. Theor. Chem. 2013, 1015, 64 10.1016/j.comptc.2013.04.009.

[ref71] Wilson P. J.; Bradley T. J.; Tozer D. J. Hybrid exchange-correlation functional determined from thermochemical data and ab initio potentials. J. Chem. Phys. 2001, 115, 9233 10.1063/1.1412605.

[ref72] Keal T. W.; Tozer D. J. Semiempirical hybrid functional with improved performance in an extensive chemical assessment. J. Chem. Phys. 2005, 123, 121103 10.1063/1.2061227. 16392467

[ref73] Becke A. D. A new mixing of Hartree-Fock and local density-functional theories. J. Chem. Phys. 1993, 98, 1372 10.1063/1.464304.

[ref74] Jin Y.; Bartlett R. J. Accurate computation of X-ray absorption spectra with ionization potential optimized global hybrid functional. J. Chem. Phys. 2018, 149, 064111 10.1063/1.5038434. 30111144

[ref75] Peverati R.; Truhlar D. G. Screened-exchange density functionals with broad accuracy for chemistry and solid-state physics. Phys. Chem. Chem. Phys. 2012, 14, 16187 10.1039/c2cp42576a. 23132141

[ref76] Yanai T.; Tew D. P.; Handy N. C. A new hybrid exchange-correlation functional using the Coulomb-attenuating method (CAM-B3LYP). Chem. Phys. Lett. 2004, 393, 51 10.1016/j.cplett.2004.06.011.

[ref77] Shao Y.; Mei Y.; Sundholm D.; Kaila V. R. I. Benchmarking the Performance of Time-Dependent Density Functional Theory Methods on Biochromophores. J. Chem. Theory Comput. 2020, 16, 587–600. 10.1021/acs.jctc.9b00823. 31815476 PMC7391796

[ref78] Verma P.; Bartlett R. J. Increasing the applicability of density functional theory. IV. Consequences of ionization-potential improved exchange-correlation potentials. J. Chem. Phys. 2014, 140, 18A534 10.1063/1.4871409. 24832342

[ref79] Jin Y.; Bartlett R. J. The QTP family of consistent functionals and potentials in Kohn-Sham density functional theory. J. Chem. Phys. 2016, 145, 034107 10.1063/1.4955497. 27448874

[ref80] Haiduke R. L. A.; Bartlett R. J. Non-empirical exchange-correlation parameterizations based on exact conditions from correlated orbital theory. J. Chem. Phys. 2018, 148, 184106 10.1063/1.5025723. 29764154

[ref81] Chai J.-D.; Head-Gordon M. Systematic optimization of long-range corrected hybrid density functionals. J. Chem. Phys. 2008, 128, 084106 10.1063/1.2834918. 18315032

[ref82] Chai J.-D.; Head-Gordon M. Long-range corrected hybrid density functionals with damped atom-atom dispersion corrections. Phys. Chem. Chem. Phys. 2008, 10, 6615–6620. 10.1039/b810189b. 18989472

[ref83] Mardirossian N.; Head-Gordon M. ωB97X-V: A 10-parameter, range-separated hybrid, generalized gradient approximation density functional with nonlocal correlation, designed by a survival-of-the-fittest strategy. Phys. Chem. Chem. Phys. 2014, 16, 9904–9924. 10.1039/c3cp54374a. 24430168

[ref84] King R. A.; Galbraith J. M.; Schaefer H. F. Negative Ion Thermochemistry: The Sulfur Fluorides SF_*n*_/SF_*n*_^–^ (n= 1–7). J. Phys. Chem. C 1996, 100, 6061–6068. 10.1021/jp9526051.

[ref85] King R. A.; Mastryukov V. S.; Schaefer H. F. The electron affinities of the silicon fluorides SiF_*n*_ (*n*=1–5). J. Chem. Phys. 1996, 105, 6880–6886. 10.1063/1.471846.

[ref86] King R. A.; Pettigrew N. D.; Schaefer H. F. The electron affinities of the perfluorocarbons C_2_F_*n*_, *n*=1–6. J. Chem. Phys. 1997, 107, 8536–8544. 10.1063/1.475005.

[ref87] Mardirossian N.; Head-Gordon M. Mapping the genome of meta-generalized gradient approximation density functionals: The search for B97M-V. J. Chem. Phys. 2015, 142, 074111 10.1063/1.4907719. 25702006

[ref88] Zhao Y.; Truhlar D. G. A new local density functional for main-group thermochemistry, transition metal bonding, thermochemical kinetics, and noncovalent interactions. J. Chem. Phys. 2006, 125, 194101 10.1063/1.2370993. 17129083

[ref89] Wang Y.; Jin X.; Yu H. S.; Truhlar D. G.; He X. Revised M06-L functional for improved accuracy on chemical reaction barrier heights, noncovalent interactions, and solid-state physics. Proc. Natl. Acad. Sci. U.S.A. 2017, 114, 8487–8492. 10.1073/pnas.1705670114. 28739954 PMC5559035

[ref90] Peverati R.; Truhlar D. G. M11-L: ALocal Density Functional That Provides Improved Accuracy for Electronic Structure Calculations in Chemistry and Physics. J. Phys. Chem. Lett. 2012, 3, 117 10.1021/jz201525m. 22910998

[ref91] Peverati R.; Truhlar D. G. An improved and broadly accurate local approximation to the exchange-correlation density functional: The MN12-L functional for electronic structure calculations in chemistry and physics. Phys. Chem. Chem. Phys. 2012, 14, 13171 10.1039/c2cp42025b. 22910998

[ref92] Yu H. S.; He X.; Truhlar D. G. MN15-L: ANew Local Exchange-Correlation Functional for Kohn-Sham Density Functional Theory with Broad Accuracy for Atoms, Molecules, and Solids. J. Chem. Theory Comput. 2016, 12, 1280–1293. 10.1021/acs.jctc.5b01082. 26722866

[ref93] Aschebrock T.; Kümmel S. Ultranonlocality and accurate band gaps from a meta-generalized gradient approximation. Phys. Rev. Res. 2019, 1, 033082 10.1103/PhysRevResearch.1.033082. 38099546

[ref94] Perdew J. P.; Wang Y. Accurate and simple analytic representation of the electron-gas correlation energy. Phys. Rev. B 1992, 45, 13244 10.1103/PhysRevB.45.13244. 10001404

[ref95] Sun J.; Perdew J. P.; Ruzsinszky A. Semilocal density functional obeying a strongly tightened bound for exchange. Proc. Natl. Acad. Sci. U.S.A. 2015, 112, 685–689. 10.1073/pnas.1423145112. 25561554 PMC4311813

[ref96] Perdew J. P.; Ruzsinszky A.; Csonka G. I.; Constantin L. A.; Sun J. Workhorse Semilocal Density Functional for Condensed Matter Physics and Quantum Chemistry. Phys. Rev. Lett. 2009, 103, 026403 10.1103/PhysRevLett.103.026403. 19659225

[ref97] Sun J.; Ruzsinszky A.; Perdew J. P. Strongly Constrained and Appropriately Normed Semilocal Density Functional. Phys. Rev. Lett. 2015, 115, 036402 10.1103/PhysRevLett.115.036402. 26230809

[ref98] Bartók A. P.; Yates J. R. Regularized SCAN functional. J. Chem. Phys. 2019, 150, 161101 10.1063/1.5094646. 31042928

[ref99] Tao J.; Perdew J. P.; Staroverov V. N.; Scuseria G. E. Climbing the Density Functional Ladder: Nonempirical Meta-Generalized Gradient Approximation Designed for Molecules and Solids. Phys. Rev. Lett. 2003, 91, 146401 10.1103/PhysRevLett.91.146401. 14611541

[ref100] Perdew J. P.; Tao J.; Staroverov V. N.; Scuseria G. E. Meta-generalized gradient approximation: Explanation of a realistic nonempirical density functional. J. Chem. Phys. 2004, 120, 6898 10.1063/1.1665298. 15267588

[ref101] Perdew J. P.; Ruzsinszky A.; Csonka G. I.; Constantin L. A.; Sun J. Erratum: Workhorse Semilocal Density Functional for Condensed Matter Physics and Quantum Chemistry [Phys. Rev. Lett. 103, 026403 (2009)]. Phys. Rev. Lett. 2011, 106, 179902 10.1103/PhysRevLett.106.179902. 19659225

[ref102] Staroverov V. N.; Scuseria G. E.; Tao J.; Perdew J. P. Comparative assessment of a new nonempirical density functional: Molecules and hydrogen-bonded complexes. J. Chem. Phys. 2003, 119, 12129 10.1063/1.1626543. 28010100

[ref103] Zhao Y.; Truhlar D. G. The M06 suite of density functionals for main group thermochemistry, thermochemical kinetics, noncovalent interactions, excited states, and transition elements: two new functionals and systematic testing of four M06-class functionals and 12 other functionals. Theor. Chem. Acc. 2008, 120, 215 10.1007/s00214-007-0310-x.

[ref104] Wang Y.; Verma P.; Jin X.; Truhlar D. G.; He X. Revised M06 density functional for main-group and transition-metal chemistry. Proc. Natl. Acad. Sci. U.S.A. 2018, 115, 10257–10262. 10.1073/pnas.1810421115. 30237285 PMC6187147

[ref105] Zhao Y.; Truhlar D. G. Exploring the Limit of Accuracy of the Global Hybrid Meta Density Functional for Main-Group Thermochemistry, Kinetics, and Noncovalent Interactions. J. Chem. Theory Comput. 2008, 4, 1849 10.1021/ct800246v. 26620329

[ref106] Yu H. S.; He X.; Li S. L.; Truhlar D. G. MN15: A Kohn-Sham global-hybrid exchange-correlation density functional with broad accuracy for multi-reference and single-reference systems and noncovalent interactions. Chem. Sci. 2016, 7, 5032–5051. 10.1039/C6SC00705H. 30155154 PMC6018516

[ref107] Peverati R.; Truhlar D. G. Improving the Accuracy of Hybrid Meta-GGA Density Functionals by Range Separation. J. Phys. Chem. Lett. 2011, 2, 2810 10.1021/jz201170d.

[ref108] Verma P.; Wang Y.; Ghosh S.; He X.; Truhlar D. G. Revised M11 Exchange-Correlation Functional for Electronic Excitation Energies and Ground-State Properties. J. Phys. Chem. A 2019, 123, 2966–2990. 10.1021/acs.jpca.8b11499. 30707029

[ref109] Mardirossian N.; Head-Gordon M. ωB97M-V: A combinatorially optimized, range-separated hybrid, meta-GGA density functional with VV10 nonlocal correlation. J. Chem. Phys. 2016, 144, 214110 10.1063/1.4952647. 27276948

[ref110] Balasubramani S. G.; Chen G. P.; Coriani S.; Diedenhofen M.; Frank M. S.; Franzke Y. J.; Furche F.; Grotjahn R.; Harding M. E.; Hättig C.; Hellweg A.; Helmich-Paris B.; Holzer C.; Huniar U.; Kaupp M.; Marefat Khah A.; Karbalaei Khani S.; Müller T.; Mack F.; Nguyen B. D.; Parker S. M.; Perlt E.; Rappoport D.; Reiter K.; Roy S.; Rückert M.; Schmitz G.; Sierka M.; Tapavicza E.; Tew D. P.; van Wüllen C.; Voora V. K.; Weigend F.; Wodyński A.; Yu J. M. TURBOMOLE: Modular program suite for ab initio quantum-chemical and condensed-matter simulations. J. Chem. Phys. 2020, 152, 184107 10.1063/5.0004635. 32414256 PMC7228783

[ref111] Dunning T. H. Gaussian basis sets for use in correlated molecular calculations. I. The atoms boron through neon and hydrogen. J. Chem. Phys. 1989, 90, 1007 10.1063/1.456153.

[ref112] Kendall R. A.; Dunning T. H.; Harrison R. J. Electron affinities of the first-row atoms revisited. Systematic basis sets and wave functions. J. Chem. Phys. 1992, 96, 6796 10.1063/1.462569.

[ref113] Woon D. E.; Dunning T. H. Gaussian basis sets for use in correlated molecular calculations. III. The atoms aluminum through argon. J. Chem. Phys. 1993, 98, 1358 10.1063/1.464303.

[ref114] Woon D. E.; Dunning T. H. Gaussian basis sets for use in correlated molecular calculations. V. Core-valence basis sets for boron through neon. J. Chem. Phys. 1995, 103, 4572 10.1063/1.470645.

[ref115] Peterson K. A.; Dunning T. H. Accurate correlation consistent basis sets for molecular core-valence correlation effects: The second row atoms Al-Ar, and the first row atoms B-Ne revisited. J. Chem. Phys. 2002, 117, 10548 10.1063/1.1520138.

[ref116] Weigend F. Accurate Coulomb-fitting basis sets for H to Rn. Phys. Chem. Chem. Phys. 2006, 8, 1057–1065. 10.1039/b515623h. 16633586

[ref117] Lehtola S.; Steigemann C.; Oliveira M. J. T.; Marques M. A. L. Recent developments in LIBXC-A comprehensive library of functionals for density functional theory. SoftwareX 2018, 7, 1–5. 10.1016/j.softx.2017.11.002.

[ref118] Ekström U.; Visscher L.; Bast R.; Thorvaldsen A. J.; Ruud K. Arbitrary-Order Density Functional Response Theory from Automatic Differentiation. J. Chem. Theory Comput. 2010, 6, 1971–1980. 10.1021/ct100117s. 26615926

[ref119] Kollwitz M.; Häser M.; Gauss J. Non-Abelian point group symmetry in direct second-order many-body perturbation theory calculations of NMR chemical shifts. J. Chem. Phys. 1998, 108, 8295–8301. 10.1063/1.476258.

[ref120] Reiter K.; Mack F.; Weigend F. Calculation of magnetic shielding constants with meta-GGA functionals employing the multipole-accelerated resolution of the identity: implementation and assessment of accuracy and efficiency. J. Chem. Theory Comput. 2018, 14, 191–197. 10.1021/acs.jctc.7b01115. 29232503

[ref121] Najibi A.; Goerigk L. The Nonlocal Kernel in van der Waals Density Functionals as an Additive Correction: An Extensive Analysis with Special Emphasis on the B97M-V and ωB97M-V Approaches. J. Chem. Theory Comput. 2018, 14, 5725–5738. 10.1021/acs.jctc.8b00842. 30299953

[ref122] Sun Q.; Zhang X.; Banerjee S.; Bao P.; Barbry M.; Blunt N. S.; Bogdanov N. A.; Booth G. H.; Chen J.; Cui Z.-H.; Eriksen J. J.; Gao Y.; Guo S.; Hermann J.; Hermes M. R.; Koh K.; Koval P.; Lehtola S.; Li Z.; Liu J.; Mardirossian N.; McClain J. D.; Motta M.; Mussard B.; Pham H. Q.; Pulkin A.; Purwanto W.; Robinson P. J.; Ronca E.; Sayfutyarova E. R.; Scheurer M.; Schurkus H. F.; Smith J. E. T.; Sun C.; Sun S.-N.; Upadhyay S.; Wagner L. K.; Wang X.; White A.; Whitfield J. D.; Williamson M. J.; Wouters S.; Yang J.; Yu J. M.; Zhu T.; Berkelbach T. C.; Sharma S.; Sokolov A. Y.; Chan G. K.-L. Recent developments in the PySCF program package. J. Chem. Phys. 2020, 153, 024109 10.1063/5.0006074. 32668948

[ref123] Frisch M. J.; Trucks G. W.; Schlegel H. B.; Scuseria G. E.; Robb M. A.; Cheeseman J. R.; Scalmani G.; Barone V.; Petersson G. A.; Nakatsuji H.; Li X.; Caricato M.; Marenich A. V.; Bloino J.; Janesko B. G.; Gomperts R.; Mennucci B.; Hratchian H. P.; Ortiz J. V.; Izmaylov A. F.; Sonnenberg J. L.; Williams-Young D.; Ding F.; Lipparini F.; Egidi F.; Goings J.; Peng B.; Petrone A.; Henderson T.; Ranasinghe D.; Zakrzewski V. G.; Gao J.; Rega N.; Zheng G.; Liang W.; Hada M.; Ehara M.; Toyota K.; Fukuda R.; Hasegawa J.; Ishida M.; Nakajima T.; Honda Y.; Kitao O.; Nakai H.; Vreven T.; Throssell K.; Montgomery J. A. Jr.; Peralta J. E.; Ogliaro F.; Bearpark M. J.; Heyd J. J.; Brothers E. N.; Kudin K. N.; Staroverov V. N.; Keith T. A.; Kobayashi R.; Normand J.; Raghavachari K.; Rendell A. P.; Burant J. C.; Iyengar S. S.; Tomasi J.; Cossi M.; Millam J. M.; Klene M.; Adamo C.; Cammi R.; Ochterski J. W.; Martin R. L.; Morokuma K.; Farkas O.; Foresman J. B.; Fox D. J. Gaussian 16, revision B.01; Gaussian Inc., 2016.

[ref124] Vahtras O.; Almlöf J.; Feyereisen M. W. Integral approximations for LCAO-SCF calculations. Chem. Phys. Lett. 1993, 213, 514–518. 10.1016/0009-2614(93)89151-7.

[ref125] Maximoff S. N.; Scuseria G. E. Nuclear magnetic resonance shielding tensors calculated with kinetic energy density-dependent exchange-correlation functionals. Chem. Phys. Lett. 2004, 390, 408–412. 10.1016/j.cplett.2004.04.049.

[ref126] Bates J. E.; Furche F. Harnessing the meta-generalized gradient approximation for time-dependent density functional theory. J. Chem. Phys. 2012, 137, 164105 10.1063/1.4759080. 23126693

[ref127] Valiev R. R.; Fliegl H.; Sundholm D. Closed-shell paramagnetic porphyrinoids. Chem. Commun. 2017, 53, 9866–9869. 10.1039/C7CC05232D. 28825092

[ref128] Valiev R. R.; Benkyi I.; Konyshev Y. V.; Fliegl H.; Sundholm D. Computational studies of aromatic and photophysical properties of expanded porphyrins. J. Phys. Chem. A 2018, 122, 4756–4767. 10.1021/acs.jpca.8b02311. 29741898

[ref129] Valiev R. R.; Baryshnikov G. V.; Nasibullin R. T.; Sundholm D.; Ågren H. When are Antiaromatic Molecules Paramagnetic. J. Phys. Chem. C 2020, 124, 21027–21035. 10.1021/acs.jpcc.0c01559.

[ref130] Magyarfalvi G.; Wolinski K.; Hinton J.; Pulay P. eMagRes; American Cancer Society, 2011, 10.1002/9780470034590.emrstm0501.pub2.

[ref131] Epstein S. T. Gauge invariance, current conservation, and GIAO’s. J. Chem. Phys. 1973, 58, 1592–1595. 10.1063/1.1679398.

[ref132] Valiev R. R.; Fliegl H.; Sundholm D. Bicycloaromaticity and Baird-type bicycloaromaticity of dithienothiophene-bridged [34]octaphyrins. Phys. Chem. Chem. Phys. 2018, 20, 17705–17713. 10.1039/C8CP03112F. 29942971

