# Correction to “Benchmarking Magnetizabilities with Recent Density Functionals”

**DOI:** 10.1021/acs.jctc.1c00466

**Published:** 2021-06-15

**Authors:** Susi Lehtola, Maria Dimitrova, Heike Fliegl, Dage Sundholm

It has been brought to our attention that our recent study^1^ erroneously employed a vanishing Hartree–Fock (HF) magnetizability for the SO_2_ molecule. The reason was a Turbomole input error for this calculation. Our analysis script did not check whether a magnetizability was found in the Gimic output, which resulted in the parsing of an empty string in our analysis, leading to the value 0.0 × 10^–30^ J/T^2^ being used in the analysis. As the correct HF magnetizability according to the procedure used in ref (1) is −301.9 × 10^–30^ J/T^2^ for SO_2_, the correct deviation from the CCSD(T) value is only 12.4 × 10^–30^ J/T^2^ instead of 301.9 × 10^–30^ J/T^2^ in the original analysis. The missing data led to an erroneous ranking of HF theory. Instead of holding the last (52nd) place, in our rectified assessment HF ranks 29th. The conclusions of the study, however, are unaffected: the magnetizabilities calculated at the HF level of theory are less accurate than those calculated with the best density functional approximations, and we cannot recommend the use of HF for magnetic properties. Rectified versions of Table 3 and Figures 1d and 4 are shown in Table 1 and Figures 1 and 2, respectively.

**Table 1:** Corrected Table 3 of Ref (1)^a^

rank	functional	MAE	ME	STD	rank	functional	MAE	ME	STD
1	BHandHLYP	3.11	2.15	4.65	27	revTPSSh	7.14	7.05	5.94
2	CAM-QTP-00	3.22	0.88	4.67	28	TPSSh	7.20	7.07	6.02
3	ωB97X-V	3.22	2.51	4.36	29	HF	7.22	–3.70	8.41
4	CAM-QTP-01	3.23	0.59	4.49	30	B97-2	7.24	7.07	6.40
5	CAM-QTP-02	3.28	–0.23	4.36	31	M08-HX	7.34	5.17	10.27
6	ωB97	3.54	2.44	4.75	32	BLYP	7.91	5.69	8.75
7	ωB97M-V	3.61	0.41	4.75	33	N12-SX	8.04	7.89	7.48
8	CAM-B3LYP	3.73	2.38	4.86	34	revTPSS	8.20	7.86	6.68
9	MN12-SX	3.80	0.22	5.34	35	TPSS	8.22	7.85	6.85
10	CAMh-B3LYP	4.23	3.22	5.17	36	revM11	8.23	6.83	10.03
11	ωB97X	4.25	3.71	5.22	37	TASK	8.27	7.31	7.43
12	QTP-17	4.58	3.77	5.45	38	BP86	8.59	7.30	8.75
13	BHLYP	4.73	0.10	6.47	39	M11-L	8.92	5.20	9.26
14	B97M-V	5.19	4.13	5.58	40	revM06	8.94	8.67	10.27
15	revB3LYP	5.45	4.34	6.13	41	PBE	9.13	7.07	9.42
16	B3LYP	5.47	4.72	5.97	42	KT3	9.19	8.38	8.08
17	MN12-L	5.79	–2.03	8.02	43	LDA	9.55	5.37	11.36
18	KT1	5.87	1.15	7.11	44	CHACHIYO	9.76	9.17	8.88
19	rSCAN	5.91	5.00	6.06	45	M11	9.93	7.61	13.77
20	PBE0	5.96	5.56	6.81	46	M06-2X	10.15	9.01	13.12
21	ωB97X-D	6.22	5.89	6.35	47	MVS	10.35	9.92	9.20
22	SCAN	6.30	5.89	5.96	48	M08-SO	10.40	8.09	14.34
23	KT2	6.42	5.58	7.21	49	N12	10.89	10.01	9.58
24	MN15-L	6.57	–5.27	6.94	50	MN15	11.45	10.45	12.82
25	B97-3	6.61	6.61	6.26	51	M06-L	12.49	12.45	9.42
26	revM06-L	7.00	6.23	5.98	52	M06	13.34	13.11	13.16

a The mean absolute errors (MAEs), mean errors (MEs), and standard deviations (STDs) for the magnetizabilities of the 27 studied molecules in units of 10^–30^ J/T^2^ from the CCSD(T) reference with the studied functionals. The functionals are ordered in increasing MAE.

**Figure 1:**
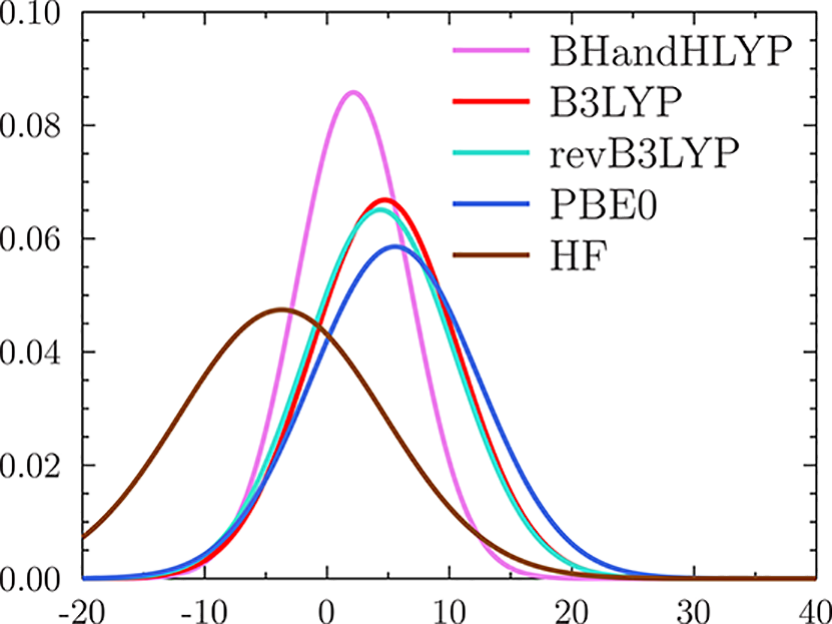
The corrected Figure 1d of ref (1) shows the normal distributions (NDs) representing the errors in the magnetizabilities for the 27 benchmarks reproduced by the studied functionals, obtained by plotting the data presented in Table 1. The curves are ordered by increasing standard deviation. The NDs at the HF level are compared to NDs obtained with a few global hybride (GH) functionals.

**Figure 2:**
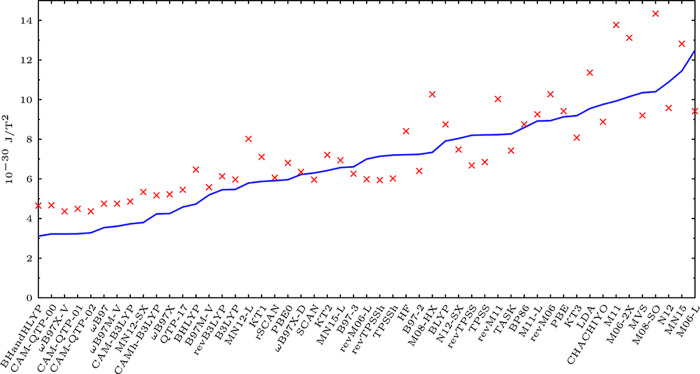
The corrected Figure 4 of ref (1) shows the mean absolute errors (blue solid line) as well as the errors’ standard deviations (red crosses) of the magnetizabilities (in 10^–30^ J/T^2^) of the 27 studied molecules obtained with the 51 functionals and at the HF level are compared to the CCSD(T) reference.

We also want to clarify the discussion in ref (1) on the magnetic gauge invariance of meta-generalized gradient (meta-GGA) functionals. Calculations of magnetizabilities using meta-GGA functionals require extensions to ensure gauge-origin independence,^2,3^ since the kinetic density (τ) depends on the gauge origin (O) in the presence of an external magnetic field.^4,5^ Recent benchmark calculations have shown that the widely used extension proposed by Maximoff and Scuseria^3^ leads to unphysical paramagnetic contributions to the nuclear magnetic shielding constants of atoms,^5,6,13^ whereas such problems do not appear when using the extension proposed by Dobson,^2^ which is commonly used for studying molecules in explicit magnetic fields^7−10^ and in some other applications.^4,11,12^ The unphysical effects are relatively small in calculations of nuclear magnetic shielding constants with many meta-GGA functionals,^5^ while calculations of magnetizabilities as the second derivative of the energy lead to significant deviations from reference data.^1^

At variance to our statement in ref (1), both TURBOMOLE and GAUSSIAN appear to use the approach of Maximoff and Scuseria^3^ for nuclear magnetic shielding calculations. However, the approach we introduced in ref (1) to compute magnetizabilities using a numerical integration of current-density susceptibilities obtained from calculations of magnetic shielding constants leads to a better gauge invariance and more accurate magnetizabilities for meta-GGA functionals than the approach based on second derivatives in combination with the approach of Maximoff and Scuseria.^3^ The approach used in TURBOMOLE has been recently discussed by Holzer et al.^6^ and Reiter et al.^13^ to which we refer for further details.
