# Supplementary material for: Benchmarking Magnetizabilities with Recent Density Functionals
Source: J Chem Theory Comput. 2021 Feb 18;17(3):1457–68. doi: 10.1021/acs.jctc.0c01190 (PMC8023670; doi:10.1021/acs.jctc.0c01190)
Supplement: Supplementary file 1 — ct0c01190_si_001.pdf [file ct0c01190_si_001.pdf]

Supporting Information: Benchmarking magnetizabilities with recent density functionals

Susi Lehtola,[*,†,‡] Maria Dimitrova,[*,†] Heike Fliegl,[*,¶] and Dage Sundholm[*,†]

†*University of Helsinki, Department of Chemistry, P.O. Box 55 (A.I. Virtanens plats 1), FI-00014 University of Helsinki, Finland*

‡*Molecular Sciences Software Institute, Blacksburg, Virginia 24061, United States*

¶*KIT, Institute of Nanotechnology, Hermann-von-Helmholtz Platz 1, D-76344 Eggenstein-Leopoldshafen, Germany*

E-mail: susi.lehtola@alumni.helsinki.fi; maria.dimitrova@helsinki.fi; heike.fliegl@kit.edu; dage.sundholm@helsinki.fi

Contents:

- Magnetically induced current-density susceptibilitise
- Calculations on tetraoxa-isophlorin
- table S2: magnetizabilities for B3LYP, B97-2, B97-3, B97M-V, and BHandHLYP
- table S3: magnetizabilities for BHLYP, BLYP, BP86, and CAM-B3LYP
- table S4: magnetizabilities for CAMh-B3LYP, CAM-QTP-00, and CAM-QTP-01
- table S5: magnetizabilities for CAM-QTP-02, CHACHIYO, HF, KT1, KT2, and KT3

- table S6: magnetizabilities for LDA, M06, M06-2X, M06-L, M08-HX, and M08-SO
- table S7: magnetizabilities for M11, M11-L, MN12-L, MN12-SX, and MN15
- table S8: magnetizabilities for MN15-L, MVS, N12, N12-SX, PBE, and PBE0
- table S9: magnetizabilities for QTP-17, revB3LYP, revM06, and revM06-L
- table S10: magnetizabilities for revM11, revTPSS, revTPSSh, rSCAN, and SCAN
- table S11: magnetizabilities for TASK, TPSS, TPSSh, ωB97, ωB97M-V, and ωB97X
- table S12: magnetizabilities for ωB97X-D, and ωB97X-V
- table S13: magnetizability errors for B3LYP, B97-2, B97-3, B97M-V, and BHandHLYP
- table S14: magnetizability errors for BHLYP, BLYP, BP86, and CAM-B3LYP
- table S15: magnetizability errors for CAMh-B3LYP, CAM-QTP-00, and CAM-QTP-01
- table S16: magnetizability errors for CAM-QTP-02, CHACHIYO, HF, KT1, KT2, and KT3
- table S17: magnetizability errors for LDA, M06, M06-2X, M06-L, M08-HX, and M08-SO
- table S18: magnetizability errors for M11, M11-L, MN12-L, MN12-SX, and MN15
- table S19: magnetizability errors for MN15-L, MVS, N12, N12-SX, PBE, and PBE0
- table S20: magnetizability errors for QTP-17, revB3LYP, revM06, and revM06-L
- table S21: magnetizability errors for revM11, revTPSS, revTPSSh, rSCAN, and SCAN
- table S22: magnetizability errors for TASK, TPSS, TPSSh, ωB97, ωB97M-V, and ωB97X

- table S23: magnetizability errors for ωB97X-D, and ωB97X-V
- table S24: comparison of Turbomole and PySCF data.

Magnetically induced current-density susceptibilities

Table S1: The expression used to calculate the magnetically induced current-density susceptibility (CDT).

$$\mathcal{J}_{\alpha}^{B_\beta} = \left.\frac{\partial J_{\alpha}^{\mathbf{B}}}{\partial B_\beta}\right|_{\mathbf{B}=\mathbf{0}} = \sum_{\mu\nu} D_{\mu\nu}\left[\frac{\partial \chi_\mu^*(\mathbf{r})}{\partial B_\beta}\frac{\partial \tilde{h}(\mathbf{r})}{\partial m_{I_\alpha}}\chi_\nu(\mathbf{r}) + \chi_\mu^*(\mathbf{r})\frac{\partial \tilde{h}(\mathbf{r})}{\partial m_{I\alpha}}\frac{\partial \chi_\nu(\mathbf{r})}{\partial B_\beta} - \sum_\gamma \epsilon_{\alpha\beta\gamma}\chi_\mu^*(\mathbf{r})\frac{\partial^2 \tilde{h}(\mathbf{r})}{\partial m_{I_\alpha}\partial B_\gamma}\chi_\nu(\mathbf{r})\right]_{\mathbf{B}=\mathbf{0}} + \sum_{\mu\nu}\left[\frac{\partial D_{\mu\nu}}{\partial B_\beta}\chi_\mu^*(\mathbf{r})\frac{\partial \tilde{h}(\mathbf{r})}{\partial m_{I_\alpha}}\chi_\nu(\mathbf{r})\right]_{\mathbf{B}=\mathbf{0}} . \quad (1)$$

The use of GIAOs eliminates the gauge origin ($\mathbf{R}_O$) from the expression we use for calculating the CDT, which is given in equation (1) in table S1. In the expression, $\mathbf{p}$ is the momentum operator, m_{I_α} are the Cartesian components (α) of the magnetic moment of nucleus I, B_β are the Cartesian components (β) of the external magnetic field, $\mathbf{D}$ is the density matrix in the atomic-orbital basis, $[\partial\mathbf{D}/\partial\mathbf{B}]_{\mathbf{B}=\mathbf{0}}$ are the magnetically perturbed density matrices, $\epsilon_{\alpha\beta\gamma}$ is the Levi–Civita symbol, $\tilde{h}(\mathbf{r})$ denotes the magnetic interaction operator without the $|\mathbf{r}-\mathbf{R}_I|^{-3}$ denominator with

$$\frac{\partial \tilde{h}(\mathbf{r})}{\partial \mathbf{m}_I} = (\mathbf{r}-\mathbf{R}_I)\times\mathbf{p} \quad (2)$$

and

$$\frac{\partial^2 \tilde{h}(\mathbf{r})}{\partial \mathbf{m}_I \partial \mathbf{B}} = \frac{1}{2}[(\mathbf{r}-\mathbf{R}_O)\cdot(\mathbf{r}-\mathbf{R}_I)\mathbf{1} - (\mathbf{r}-\mathbf{R}_O)(\mathbf{r}-\mathbf{R}_I)], \quad (3)$$

and $\mathbf{R}_I$ is the position of nucleus I. All terms that contain the gauge origin $\mathbf{R}_O$ cancel in equation (1), making the CDT calculation independent of the gauge origin; this is demonstrated in figure S1 for a different choice of the gauge origin. All terms containing the nuclear position $\mathbf{R}_I$ also cancel, eliminating explicit references to the nuclear coordinates.

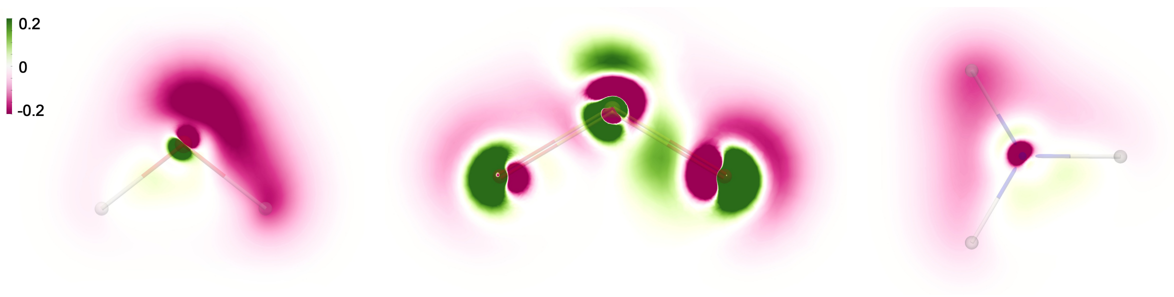

(a) (b) (c)

Figure S1: Visualization of the isotropic magnetizability density $\overline{\rho}^{\xi}(\mathbf{r})$ shown in the molecular plane of H_2O S1(a) and SO_2 S1(b) as well as in the plane formed by the hydrogen atoms of NH_3 S1(c), positioned $0.06\,a_0$ away from the N atom towards the hydrogen atoms. Negative contributions are shown in pink, and positive ones in green. The gauge origin $\mathbf{R}_O$ is $(1, 1, 1)\,a_0$.

Calculations on tetraoxa-isophlorin

Valiev et al.[S1] found the isotropic magnetizability of tetraoxa-isophlorin (molecule V in their work) to be 15.8 a.u. at the LMP2/cc-pVDZ level of theory [local second-order Møller–Plesset perturbation theory], while B3LYP/def2-TZVP calculations yielded a value of 65.9 a.u., which is over four times the LMP2 value. Repeating the calculations of Valiev et al.[S1] with the present approach using the def2-TZVP basis set, we obtained a magnetizability of 65.2 a.u. at the B3LYP level, which agrees within 1% with the value of Valiev et al.[S1]; this difference can be tentatively attributed to the use of density fitting in the present work. MN12-SX, which has no exact exchange in the long range, predicts a susceptibility of 63.4 a.u., which is four times larger than the LMP2/cc-pVDZ value and close to the B3LYP value. Functionals with no exact exchange like PBE yield even larger values, > 100 a.u. In contrast, calculations at the CAM-B3LYP level with 65% LR HF exchange yield a magnetizability of 20.7 a.u., which agrees well with the LMP2 reference value. BHandHLYP contains 50% LR HF exchange, and yields a magnetizability of 23.7 a.u., which is also in qualitative agreement with LMP2. Range-separated functionals with 100% LR HF exchange like ωB97X and ωB97 yield a magnetizability for tetraoxa-isophlorin that is close to zero or even negative like the HF value, which is -11.6 a.u.[S1] The B3LYP/def2-TZVP optimized geometry of ref. S1,

attached here in xyz format, was used in the calculations on tetraoxa-isophlorin.

Table S2: Magnetizabilities in units of 10^{-30}J/T^2 for the B3LYP, B97-2, B97-3, B97M-V, and BHandHLYP functionals in the aug-cc-pCVQZ basis set from calculations with Turbomole and Gimic compared to CCSD(T) data from ref. S2.

Molecule	B3LYP	B97-2	B97-3	B97M-V	BHandHLYP	CCSD(T)
AlF	-396.5	-393.4	-392.6	-396.1	-395.6	-394.5
C_2H_4	-336.7	-334.3	-334.6	-334.8	-343.0	-345.6
C_3H_4	-463.1	-462.6	-464.1	-460.6	-468.8	-478.9
CH_2O	-114.9	-116.6	-115.4	-132.8	-123.8	-127.4
CH_3F	-312.4	-312.2	-313.3	-309.4	-314.9	-315.7
CH_4	-317.0	-314.6	-314.6	-313.2	-315.7	-316.9
CO	-206.6	-202.5	-201.2	-208.5	-205.0	-209.5
FCCH	-440.1	-438.3	-439.2	-440.5	-443.6	-441.6
FCN	-367.4	-365.4	-365.9	-368.3	-370.5	-370.0
H_2C_2O	-422.1	-421.2	-421.0	-425.9	-425.0	-423.9
H_2O	-236.7	-233.5	-233.9	-233.4	-234.0	-235.1
H_2S	-455.1	-452.0	-452.4	-452.3	-453.5	-455.1
H_4C_2O	-526.9	-527.3	-529.0	-519.7	-534.5	-535.2
HCN	-269.4	-265.0	-265.4	-268.9	-272.7	-271.8
HCP	-487.4	-481.9	-482.9	-485.9	-494.3	-492.8
HF	-178.4	-175.9	-176.2	-176.1	-175.8	-176.4
HFCO	-300.5	-298.0	-298.0	-302.5	-304.0	-307.2
HOF	-231.1	-230.7	-232.2	-231.5	-236.7	-235.4
LiF	-194.7	-193.4	-194.5	-195.6	-192.6	-195.5
LiH	-130.8	-129.6	-127.0	-132.5	-126.4	-127.2
N_2	-202.0	-197.2	-197.3	-203.2	-201.6	-205.2
N_2O	-333.8	-332.6	-334.1	-333.0	-336.7	-339.1
NH_3	-291.2	-287.9	-288.4	-287.0	-289.3	-290.3
O_3	238.7	239.4	264.0	99.3	336.6	121.5
OCS	-579.6	-577.2	-578.8	-577.6	-585.6	-584.1
OF_2	-234.1	-235.2	-238.2	-240.0	-250.2	-247.1
PN	-292.2	-285.4	-284.3	-302.0	-295.5	-308.2
SO_2	-296.1	-289.5	-290.9	-301.3	-296.6	-314.3

Table S3: Magnetizabilities in units of 10^{-30}J/T^2 for the BHLYP, BLYP, BP86, and CAM-B3LYP functionals in the aug-cc-pCVQZ basis set from calculations with TURBOMOLE and GIMIC compared to CCSD(T) data from ref. S2.

Molecule	BHLYP	BLYP	BP86	CAM-B3LYP	CCSD(T)
AlF	-397.1	-399.2	-394.3	-397.0	-394.5
C_2H_4	-343.5	-333.4	-331.0	-339.4	-345.6
C_3H_4	-473.1	-458.4	-460.1	-468.1	-478.9
CH_2O	-114.1	-109.3	-108.1	-115.3	-127.4
CH_3F	-319.0	-309.5	-311.4	-314.6	-315.7
CH_4	-325.2	-318.1	-318.5	-320.0	-316.9
CO	-205.9	-209.1	-205.2	-208.4	-209.5
FCCH	-445.4	-438.5	-437.9	-441.8	-441.6
FCN	-371.6	-366.4	-365.0	-369.5	-370.0
H_2C_2O	-431.0	-420.7	-422.0	-424.8	-423.9
H_2O	-236.6	-239.4	-237.5	-237.5	-235.1
H_2S	-462.6	-457.0	-456.6	-456.7	-455.1
H_4C_2O	-542.1	-520.4	-523.5	-531.3	-535.2
HCN	-272.9	-268.7	-264.4	-272.0	-271.8
HCP	-492.9	-485.6	-479.2	-488.6	-492.8
HF	-177.0	-181.0	-179.3	-179.0	-176.4
HFCO	-303.8	-299.4	-296.5	-302.9	-307.2
HOF	-238.8	-226.7	-227.8	-233.4	-235.4
LiF	-193.1	-196.3	-197.0	-195.6	-195.5
LiH	-129.4	-136.5	-133.2	-129.3	-127.2
N_2	-202.4	-203.7	-199.6	-204.3	-205.2
N_2O	-338.8	-332.0	-332.6	-336.0	-339.1
NH_3	-294.3	-293.4	-291.9	-292.7	-290.3
O_3	356.9	180.1	180.9	258.1	121.5
OCS	-588.0	-576.1	-575.0	-583.4	-584.1
OF_2	-251.5	-220.6	-222.1	-239.8	-247.1
PN	-293.9	-292.4	-284.7	-297.4	-308.2
SO_2	-297.0	-298.4	-292.7	-300.7	-314.3

Table S4: Magnetizabilities in units of 10^{-30}J/T^2 for the CAMh-B3LYP, CAM-QTP-00, and CAM-QTP-01 functionals in the aug-cc-pCVQZ basis set from calculations with TURBOMOLE and GIMIC compared to CCSD(T) data from ref. S2.

Molecule	CAMh-B3LYP	CAM-QTP-00	CAM-QTP-01	CCSD(T)
AlF	-396.9	-394.5	-397.2	-394.5
C_2H_4	-338.5	-344.7	-341.6	-345.6
C_3H_4	-466.2	-472.4	-471.9	-478.9
CH_2O	-115.6	-124.5	-115.3	-127.4
CH_3F	-313.6	-316.7	-316.6	-315.7
CH_4	-318.6	-317.5	-322.7	-316.9
CO	-207.9	-205.2	-209.4	-209.5
FCCH	-441.2	-444.8	-443.5	-441.6
FCN	-368.8	-371.8	-371.2	-370.0
H_2C_2O	-423.6	-427.0	-427.4	-423.9
H_2O	-237.2	-234.0	-238.0	-235.1
H_2S	-455.8	-454.0	-458.5	-455.1
H_4C_2O	-529.5	-538.2	-535.6	-535.2
HCN	-271.2	-273.9	-273.9	-271.8
HCP	-488.4	-494.5	-489.8	-492.8
HF	-178.8	-175.5	-179.1	-176.4
HFCO	-302.2	-305.3	-304.5	-307.2
HOF	-232.5	-238.7	-235.6	-235.4
LiF	-195.4	-192.6	-195.8	-195.5
LiH	-129.8	-124.9	-128.6	-127.2
N_2	-203.6	-202.3	-205.6	-205.2
N_2O	-335.2	-338.2	-337.7	-339.1
NH_3	-292.0	-289.8	-293.9	-290.3
O_3	250.1	373.1	283.2	121.5
OCS	-582.1	-588.0	-586.4	-584.1
OF_2	-237.7	-255.0	-244.9	-247.1
PN	-295.9	-297.6	-300.3	-308.2
SO_2	-298.8	-298.6	-303.1	-314.3

Table S5: Magnetizabilities in units of 10^{-30}J/T^2 for the CAM-QTP-02, CHACHIYO, HF, KT1, KT2, and KT3 functionals in the aug-cc-pCVQZ basis set from calculations with TURBOMOLE and GIMIC compared to CCSD(T) data from ref. S2.

Molecule	CAM-QTP-02	CHACHIYO	HF	KT1	KT2	KT3	CCSD(T)
AlF	-397.4	-392.2	-399.2	-398.4	-392.4	-394.2	-394.5
C_2H_4	-343.0	-329.0	-354.8	-338.6	-335.2	-332.4	-345.6
C_3H_4	-473.3	-458.7	-478.1	-461.5	-457.3	-453.2	-478.9
CH_2O	-116.5	-109.3	-139.4	-116.8	-118.0	-117.9	-127.4
CH_3F	-317.4	-310.8	-317.9	-309.8	-307.4	-305.3	-315.7
CH_4	-323.3	-315.5	-313.6	-320.7	-316.0	-311.8	-316.9
CO	-209.3	-202.6	-204.5	-214.0	-209.1	-206.1	-209.5
FCCH	-444.5	-436.3	-452.2	-445.0	-440.2	-437.0	-441.6
FCN	-372.1	-363.4	-378.0	-372.4	-367.6	-365.1	-370.0
H_2C_2O	-428.6	-419.6	-432.6	-428.1	-422.1	-416.9	-423.9
H_2O	-237.8	-235.9	-231.2	-238.8	-235.0	-233.8	-235.1
H_2S	-459.1	-453.4	-452.6	-462.1	-455.7	-450.8	-455.1
H_4C_2O	-537.8	-522.2	-544.9	-527.0	-521.3	-516.5	-535.2
HCN	-274.8	-261.7	-280.1	-274.8	-270.5	-267.1	-271.8
HCP	-491.2	-475.5	-511.6	-493.6	-487.9	-483.9	-492.8
HF	-178.8	-178.4	-172.7	-179.8	-176.8	-176.5	-176.4
HFCO	-305.5	-294.7	-311.5	-303.3	-299.0	-297.5	-307.2
HOF	-237.1	-227.2	-244.6	-231.4	-227.6	-224.9	-235.4
LiF	-195.5	-196.1	-190.7	-199.1	-196.1	-193.8	-195.5
LiH	-128.0	-131.7	-125.3	-139.1	-137.1	-138.0	-127.2
N_2	-205.9	-197.0	-202.9	-209.8	-205.0	-201.2	-205.2
N_2O	-338.6	-331.6	-342.8	-334.6	-330.5	-328.0	-339.1
NH_3	-294.1	-289.6	-287.4	-293.9	-289.6	-287.0	-290.3
O_3	303.8	183.6	578.9	131.9	138.6	149.2	121.5
OCS	-587.9	-573.1	-597.5	-582.1	-575.6	-571.6	-584.1
OF_2	-248.4	-222.1	-271.8	-231.7	-226.4	-223.3	-247.1
PN	-300.9	-279.9	-304.2	-302.1	-297.0	-291.1	-308.2
SO_2	-303.7	-288.9	0.0	-304.6	-297.1	-292.6	-314.3

Table S6: Magnetizabilities in units of 10^{-30}J/T^2 for the LDA, M06, M06-2X, M06-L, M08-HX, and M08-SO functionals in the aug-cc-pCVQZ basis set from calculations with Turbomole and Gimic compared to CCSD(T) data from ref. S2.

Molecule	LDA	M06	M06-2X	M06-L	M08-HX	M08-SO	CCSD(T)
AlF	-395.8	-387.1	-392.9	-382.7	-397.2	-395.8	-394.5
C_2H_4	-331.1	-332.4	-329.9	-327.1	-331.2	-334.3	-345.6
C_3H_4	-464.4	-465.3	-461.4	-461.5	-462.7	-463.7	-478.9
CH_2O	-95.9	-104.5	-94.1	-123.6	-94.1	-89.4	-127.4
CH_3F	-315.4	-313.4	-317.2	-311.1	-319.5	-319.0	-315.7
CH_4	-329.4	-316.3	-319.9	-309.4	-322.6	-323.0	-316.9
CO	-206.6	-192.3	-193.9	-195.3	-201.7	-195.2	-209.5
FCCH	-438.6	-434.5	-439.7	-434.0	-442.4	-441.0	-441.6
FCN	-365.3	-358.7	-364.6	-359.9	-368.3	-365.2	-370.0
H_2C_2O	-427.7	-417.3	-418.1	-418.8	-423.1	-419.3	-423.9
H_2O	-240.9	-233.1	-235.6	-230.2	-235.8	-236.7	-235.1
H_2S	-466.0	-451.7	-457.7	-447.1	-457.1	-459.1	-455.1
H_4C_2O	-529.8	-529.4	-540.3	-521.1	-543.4	-543.5	-535.2
HCN	-265.1	-252.4	-260.4	-252.6	-265.2	-262.4	-271.8
HCP	-477.5	-465.0	-476.4	-462.9	-483.3	-480.5	-492.8
HF	-181.1	-175.1	-176.6	-173.8	-177.0	-177.5	-176.4
HFCO	-296.9	-292.1	-292.9	-292.4	-295.7	-293.5	-307.2
HOF	-229.0	-228.3	-235.1	-230.0	-236.4	-235.3	-235.4
LiF	-196.3	-190.4	-193.4	-191.8	-193.2	-193.0	-195.5
LiH	-136.0	-130.3	-128.1	-127.7	-129.1	-129.6	-127.2
N_2	-201.1	-181.2	-189.1	-186.5	-195.7	-189.7	-205.2
N_2O	-334.3	-326.3	-332.8	-329.0	-335.7	-331.7	-339.1
NH_3	-298.1	-288.2	-291.9	-283.0	-292.0	-293.4	-290.3
O_3	195.2	413.4	492.9	156.2	348.2	647.4	121.5
OCS	-576.6	-570.7	-578.3	-569.4	-583.5	-580.2	-584.1
OF_2	-220.3	-228.5	-242.9	-234.3	-247.0	-241.6	-247.1
PN	-284.6	-249.4	-259.9	-267.0	-283.7	-259.9	-308.2
SO_2	-295.1	-276.3	-277.4	-285.4	-287.7	-271.8	-314.3

Table S7: Magnetizabilities in units of 10^{-30}J/T^2 for the M11, M11-L, MN12-L, MN12-SX, and MN15 functionals in the aug-cc-pCVQZ basis set from calculations with Turbomole and Gimic compared to CCSD(T) data from ref. S2.

Molecule	M11	M11-L	MN12-L	MN12-SX	MN15	CCSD(T)
AlF	-391.5	-403.1	-407.1	-403.4	-400.2	-394.5
C_2H_4	-331.3	-334.1	-340.4	-338.4	-330.4	-345.6
C_3H_4	-466.6	-461.3	-471.1	-467.1	-460.0	-478.9
CH_2O	-89.0	-135.4	-145.0	-128.4	-91.0	-127.4
CH_3F	-320.7	-307.8	-313.8	-315.5	-314.9	-315.7
CH_4	-323.7	-312.0	-315.6	-317.7	-319.4	-316.9
CO	-199.2	-203.4	-211.4	-207.8	-194.3	-209.5
FCCH	-440.0	-444.9	-446.6	-445.2	-437.2	-441.6
FCN	-365.7	-368.1	-371.7	-370.9	-361.1	-370.0
H_2C_2O	-424.3	-431.4	-434.6	-428.8	-416.3	-423.9
H_2O	-236.4	-227.8	-230.5	-233.4	-235.1	-235.1
H_2S	-459.1	-450.6	-454.1	-454.8	-454.8	-455.1
H_4C_2O	-545.2	-518.5	-525.5	-532.5	-531.5	-535.2
HCN	-262.7	-263.9	-273.5	-271.6	-259.8	-271.8
HCP	-471.9	-489.0	-501.5	-494.9	-481.5	-492.8
HF	-177.9	-169.8	-173.5	-175.2	-176.8	-176.4
HFCO	-293.7	-300.3	-305.4	-303.3	-289.1	-307.2
HOF	-234.8	-228.6	-234.8	-237.4	-230.1	-235.4
LiF	-194.0	-188.3	-191.3	-190.8	-195.5	-195.5
LiH	-126.4	-150.0	-145.0	-140.5	-131.5	-127.2
N_2	-193.7	-193.7	-204.3	-201.3	-186.9	-205.2
N_2O	-334.5	-330.7	-336.1	-336.1	-328.4	-339.1
NH_3	-292.7	-284.3	-286.3	-289.5	-291.0	-290.3
O_3	484.4	84.9	45.6	131.1	571.0	121.5
OCS	-582.9	-579.9	-587.3	-586.0	-574.3	-584.1
OF_2	-241.3	-242.3	-249.8	-253.4	-230.3	-247.1
PN	-266.2	-293.5	-330.2	-311.0	-271.6	-308.2
SO_2	-273.4	-290.9	-312.3	-303.3	-268.9	-314.3

Table S8: Magnetizabilities in units of 10^{-30}J/T^2 for the MN15-L, MVS, N12, N12-SX, PBE, and PBE0 functionals in the aug-cc-pCVQZ basis set from calculations with TURBOMOLE and GIMIC compared to CCSD(T) data from ref. S2.

Molecule	MN15-L	MVS	N12	N12-SX	PBE	PBE0	CCSD(T)
AlF	-410.2	-384.3	-394.1	-394.7	-396.9	-394.3	-394.5
C_2H_4	-343.2	-319.8	-331.1	-333.8	-330.8	-335.3	-345.6
C_3H_4	-470.4	-459.6	-456.3	-463.8	-459.5	-465.1	-478.9
CH_2O	-142.4	-123.5	-112.9	-113.3	-104.9	-112.8	-127.4
CH_3F	-314.1	-309.1	-307.8	-313.8	-311.2	-314.4	-315.7
CH_4	-318.8	-315.1	-315.6	-316.5	-320.3	-318.4	-316.9
CO	-215.3	-198.0	-205.2	-201.1	-205.6	-202.9	-209.5
FCCH	-450.2	-436.9	-433.4	-436.2	-437.6	-439.9	-441.6
FCN	-376.7	-365.7	-360.8	-362.6	-365.0	-366.6	-370.0
H_2C_2O	-438.1	-428.8	-416.6	-419.6	-421.8	-423.6	-423.9
H_2O	-235.8	-232.3	-234.3	-233.7	-238.5	-235.2	-235.1
H_2S	-459.0	-456.0	-451.4	-453.9	-458.6	-456.2	-455.1
H_4C_2O	-530.1	-519.8	-516.6	-528.2	-523.9	-531.5	-535.2
HCN	-277.0	-256.3	-265.3	-264.0	-264.4	-266.0	-271.8
HCP	-508.5	-467.7	-477.2	-480.1	-479.3	-482.7	-492.8
HF	-176.9	-174.8	-176.2	-176.0	-180.1	-177.1	-176.4
HFCO	-309.6	-297.4	-294.8	-295.6	-296.8	-298.7	-307.2
HOF	-239.5	-226.7	-222.6	-230.0	-227.4	-232.8	-235.4
LiF	-197.3	-194.5	-191.7	-193.3	-196.2	-194.1	-195.5
LiH	-137.9	-125.5	-139.1	-128.9	-135.2	-129.1	-127.2
N_2	-208.7	-191.9	-199.8	-195.5	-199.8	-198.2	-205.2
N_2O	-340.0	-332.9	-326.5	-329.9	-331.8	-334.2	-339.1
NH_3	-291.9	-287.4	-288.9	-288.6	-293.1	-290.4	-290.3
O_3	63.6	136.6	185.3	292.9	183.4	257.6	121.5
OCS	-590.7	-576.0	-568.4	-576.7	-574.8	-579.6	-584.1
OF_2	-255.0	-228.6	-217.6	-233.7	-221.6	-238.3	-247.1
PN	-330.5	-277.7	-285.9	-283.2	-284.3	-285.0	-308.2
SO_2	-318.4	-289.8	-283.4	-284.2	-293.8	-291.5	-314.3

Table S9: Magnetizabilities in units of 10^{-30}J/T^2 for the QTP-17, revB3LYP, revM06, and revM06-L functionals in the aug-cc-pCVQZ basis set from calculations with TURBOMOLE and GIMIC compared to CCSD(T) data from ref. S2.

Molecule	QTP-17	revB3LYP	revM06	revM06-L	CCSD(T)
AlF	-397.4	-396.9	-389.7	-385.9	-394.5
C_2H_4	-338.5	-337.0	-329.6	-326.6	-345.6
C_3H_4	-464.1	-463.6	-462.6	-461.2	-478.9
CH_2O	-116.8	-113.8	-104.4	-134.0	-127.4
CH_3F	-312.7	-312.8	-315.2	-310.5	-315.7
CH_4	-316.9	-318.3	-318.4	-312.0	-316.9
CO	-207.1	-206.8	-197.2	-206.4	-209.5
FCCH	-441.1	-440.4	-438.2	-437.8	-441.6
FCN	-368.4	-367.7	-364.2	-366.5	-370.0
H_2C_2O	-422.8	-422.9	-421.1	-427.7	-423.9
H_2O	-236.6	-237.1	-234.7	-230.8	-235.1
H_2S	-455.3	-456.3	-456.3	-446.9	-455.1
H_4C_2O	-528.2	-527.8	-533.4	-517.3	-535.2
HCN	-270.8	-269.6	-261.3	-264.7	-271.8
HCP	-490.0	-487.5	-476.1	-478.4	-492.8
HF	-178.2	-178.6	-176.2	-174.4	-176.4
HFCO	-301.7	-300.7	-294.4	-298.9	-307.2
HOF	-232.2	-231.4	-232.7	-231.8	-235.4
LiF	-194.6	-194.8	-194.0	-195.1	-195.5
LiH	-130.9	-131.3	-127.8	-123.9	-127.2
N_2	-202.7	-202.2	-191.7	-200.5	-205.2
N_2O	-334.4	-334.1	-333.2	-334.6	-339.1
NH_3	-291.2	-292.0	-290.6	-284.5	-290.3
O_3	251.4	239.5	395.7	93.9	121.5
OCS	-581.2	-580.0	-578.1	-577.1	-584.1
OF_2	-237.2	-234.2	-238.1	-241.0	-247.1
PN	-294.2	-292.3	-269.8	-305.2	-308.2
SO_2	-297.1	-296.4	-280.9	-301.8	-314.3

Table S10: Magnetizabilities in units of 10^{-30}J/T^2 for the revM11, revTPSS, revTPSSh, rSCAN, and SCAN functionals in the aug-cc-pCVQZ basis set from calculations with Turbomole and Gimic compared to CCSD(T) data from ref. S2.

Molecule	revM11	revTPSS	revTPSSh	rSCAN	SCAN	CCSD(T)
AlF	-393.5	-393.2	-392.7	-395.0	-392.0	-394.5
C_2H_4	-333.0	-332.0	-333.7	-332.3	-333.0	-345.6
C_3H_4	-467.4	-457.7	-460.2	-463.4	-462.4	-478.9
CH_2O	-99.7	-123.2	-124.6	-121.9	-126.4	-127.4
CH_3F	-316.8	-308.9	-310.3	-311.8	-310.4	-315.7
CH_4	-320.1	-308.8	-309.3	-318.5	-314.4	-316.9
CO	-201.9	-204.9	-204.1	-206.7	-206.9	-209.5
FCCH	-437.8	-436.7	-437.8	-439.2	-438.3	-441.6
FCN	-365.4	-365.2	-365.9	-366.6	-365.8	-370.0
H_2C_2O	-418.7	-416.3	-417.7	-427.3	-426.0	-423.9
H_2O	-238.1	-235.6	-234.6	-235.0	-234.2	-235.1
H_2S	-456.2	-448.5	-448.7	-457.5	-453.9	-455.1
H_4C_2O	-538.2	-520.7	-523.9	-524.6	-519.8	-535.2
HCN	-264.1	-264.8	-265.5	-266.4	-265.3	-271.8
HCP	-473.1	-481.1	-482.5	-482.5	-481.7	-492.8
HF	-179.9	-178.5	-177.5	-176.8	-176.5	-176.4
HFCO	-298.1	-298.0	-298.9	-298.8	-299.3	-307.2
HOF	-233.8	-230.3	-232.2	-230.1	-230.9	-235.4
LiF	-196.9	-196.2	-195.3	-195.9	-195.6	-195.5
LiH	-123.4	-128.5	-127.2	-130.9	-130.4	-127.2
N_2	-195.5	-199.0	-198.7	-201.4	-199.8	-205.2
N_2O	-334.0	-331.3	-332.3	-333.6	-332.6	-339.1
NH_3	-293.0	-287.5	-287.1	-290.3	-288.7	-290.3
O_3	445.5	142.5	167.9	138.7	143.4	121.5
OCS	-580.8	-573.6	-575.8	-578.0	-577.7	-584.1
OF_2	-239.0	-231.2	-236.8	-232.4	-234.3	-247.1
PN	-274.0	-288.1	-288.6	-294.8	-292.3	-308.2
SO_2	-287.3	-291.7	-291.5	-297.5	-296.4	-314.3

Table S11: Magnetizabilities in units of 10^{-30}J/T^2 for the TASK, TPSS, TPSSh, ωB97, ωB97M-V, and ωB97X functionals in the aug-cc-pCVQZ basis set from calculations with TURBOMOLE and GIMIC compared to CCSD(T) data from ref. S2.

Molecule	TASK	TPSS	TPSSh	ωB97	ωB97M-V	ωB97X	CCSD(T)
AlF	-383.3	-393.9	-393.3	-397.0	-400.1	-395.5	-394.5
C_2H_4	-325.4	-332.1	-333.8	-338.1	-340.5	-337.5	-345.6
C_3H_4	-460.8	-458.5	-460.9	-471.5	-469.9	-469.6	-478.9
CH_2O	-132.9	-120.0	-121.7	-118.9	-115.8	-116.4	-127.4
CH_3F	-309.3	-309.5	-310.9	-315.3	-315.0	-314.9	-315.7
CH_4	-310.7	-311.6	-311.8	-318.7	-321.0	-317.9	-316.9
CO	-204.5	-204.7	-203.9	-208.2	-210.5	-205.8	-209.5
FCCH	-439.1	-436.7	-437.8	-442.1	-444.6	-441.2	-441.6
FCN	-367.8	-364.8	-365.5	-370.3	-371.8	-368.5	-370.0
H_2C_2O	-431.3	-417.9	-419.1	-427.0	-427.2	-424.6	-423.9
H_2O	-231.2	-235.7	-234.7	-235.9	-237.3	-235.1	-235.1
H_2S	-454.3	-450.6	-450.5	-454.2	-458.2	-454.3	-455.1
H_4C_2O	-519.1	-521.3	-524.5	-533.9	-533.9	-532.5	-535.2
HCN	-262.1	-264.4	-265.1	-269.5	-274.4	-268.6	-271.8
HCP	-478.2	-480.5	-481.9	-481.9	-491.0	-483.8	-492.8
HF	-173.7	-178.4	-177.3	-178.4	-178.7	-177.2	-176.4
HFCO	-298.6	-297.8	-298.6	-303.9	-304.0	-301.0	-307.2
HOF	-231.1	-229.3	-231.3	-234.5	-235.3	-233.5	-235.4
LiF	-195.1	-195.3	-194.6	-197.9	-196.3	-197.2	-195.5
LiH	-120.4	-129.5	-128.0	-126.4	-133.9	-129.3	-127.2
N_2	-197.7	-198.8	-198.4	-202.4	-207.1	-200.6	-205.2
N_2O	-335.7	-330.8	-331.8	-337.7	-337.5	-336.0	-339.1
NH_3	-286.1	-288.4	-287.8	-290.3	-292.8	-289.9	-290.3
O_3	142.9	151.0	176.9	236.9	225.4	260.5	121.5
OCS	-580.8	-573.6	-575.8	-585.6	-586.3	-583.2	-584.1
OF_2	-236.8	-228.9	-234.8	-243.4	-245.4	-241.1	-247.1
PN	-289.1	-287.3	-287.7	-294.6	-300.7	-292.2	-308.2
SO_2	-291.5	-291.9	-291.6	-300.8	-303.8	-296.5	-314.3

Table S12: Magnetizabilities in units of 10^{-30}J/T^2 for the ωB97X-D, and ωB97X-V functionals in the aug-cc-pCVQZ basis set from calculations with TURBOMOLE and GIMIC compared to CCSD(T) data from ref. S2.

Molecule	ωB97X-D	ωB97X-V	CCSD(T)
AlF	-393.1	-396.5	-394.5
C_2H_4	-335.5	-338.9	-345.6
C_3H_4	-466.9	-469.9	-478.9
CH_2O	-114.7	-118.6	-127.4
CH_3F	-314.1	-314.9	-315.7
CH_4	-316.1	-317.7	-316.9
CO	-202.4	-207.4	-209.5
FCCH	-439.8	-442.4	-441.6
FCN	-366.2	-370.1	-370.0
H_2C_2O	-422.1	-425.3	-423.9
H_2O	-233.8	-236.0	-235.1
H_2S	-452.9	-454.2	-455.1
H_4C_2O	-530.1	-532.8	-535.2
HCN	-266.2	-270.7	-271.8
HCP	-482.7	-486.5	-492.8
HF	-175.9	-178.1	-176.4
HFCO	-297.9	-303.1	-307.2
HOF	-232.0	-234.7	-235.4
LiF	-195.6	-196.4	-195.5
LiH	-131.5	-127.4	-127.2
N_2	-197.7	-202.9	-205.2
N_2O	-334.3	-336.8	-339.1
NH_3	-288.7	-290.5	-290.3
O_3	267.2	251.2	121.5
OCS	-579.6	-584.7	-584.1
OF_2	-238.0	-243.8	-247.1
PN	-286.5	-296.0	-308.2
SO_2	-290.6	-299.8	-314.3

Table S13: Deviations of the magnetizabilities computed with TURBOMOLE and GIMIC for the B3LYP, B97-2, B97-3, B97M-V, and BHandHLYP functionals in the aug-cc-pCVQZ basis set from CCSD(T) data from ref. S2 in units of 10^{-30}J/T^2.

Molecule	B3LYP	B97-2	B97-3	B97M-V	BHandHLYP
AlF	-2.0	1.1	1.9	-1.6	-1.1
C_2H_4	8.9	11.3	11.0	10.8	2.6
C_3H_4	15.8	16.3	14.8	18.3	10.1
CH_2O	12.5	10.8	12.0	-5.4	3.6
CH_3F	3.3	3.5	2.4	6.3	0.8
CH_4	-0.1	2.3	2.3	3.7	1.2
CO	2.9	7.0	8.3	1.0	4.5
FCCH	1.5	3.3	2.4	1.1	-2.0
FCN	2.6	4.6	4.1	1.7	-0.5
H_2C_2O	1.8	2.7	2.9	-2.0	-1.1
H_2O	-1.6	1.6	1.2	1.7	1.1
H_2S	-0.0	3.1	2.7	2.8	1.6
H_4C_2O	8.3	7.9	6.2	15.5	0.7
HCN	2.4	6.8	6.4	2.9	-0.9
HCP	5.4	10.9	9.9	6.9	-1.5
HF	-2.0	0.5	0.2	0.3	0.6
HFCO	6.7	9.2	9.2	4.7	3.2
HOF	4.3	4.7	3.2	3.9	-1.3
LiF	0.8	2.1	1.0	-0.1	2.9
LiH	-3.6	-2.4	0.2	-5.3	0.8
N_2	3.2	8.0	7.9	2.0	3.6
N_2O	5.3	6.5	5.0	6.1	2.4
NH_3	-0.9	2.4	1.9	3.3	1.0
O_3	117.2	117.9	142.5	-22.2	215.1
OCS	4.5	6.9	5.3	6.5	-1.5
OF_2	13.0	11.9	8.9	7.1	-3.1
PN	16.0	22.8	23.9	6.2	12.7
SO_2	18.2	24.8	23.4	13.0	17.7
MAE*	5.5	7.2	6.6	5.2	3.1
ME*	4.7	7.1	6.6	4.1	2.2
STD*	6.0	6.4	6.3	5.6	4.7

* Statistics for the mean absolute error (MAE), mean error (ME) and the standard deviation (STD) exclude O_3.

Table S14: Deviations of the magnetizabilities computed with TURBOMOLE and GIMIC for the BHLYP, BLYP, BP86, and CAM-B3LYP functionals in the aug-cc-pCVQZ basis set from CCSD(T) data from ref. S2 in units of 10^{-30}J/T^2.

Molecule	BHLYP	BLYP	BP86	CAM-B3LYP
AlF	-2.6	-4.7	0.2	-2.5
C_2H_4	2.1	12.2	14.6	6.2
C_3H_4	5.8	20.5	18.8	10.8
CH_2O	13.3	18.1	19.3	12.1
CH_3F	-3.3	6.2	4.3	1.1
CH_4	-8.3	-1.2	-1.6	-3.1
CO	3.6	0.4	4.3	1.1
FCCH	-3.8	3.1	3.7	-0.2
FCN	-1.6	3.6	5.0	0.5
H_2C_2O	-7.1	3.2	1.9	-0.9
H_2O	-1.5	-4.3	-2.4	-2.4
H_2S	-7.5	-1.9	-1.5	-1.6
H_4C_2O	-6.9	14.8	11.7	3.9
HCN	-1.1	3.1	7.4	-0.2
HCP	-0.1	7.2	13.6	4.2
HF	-0.6	-4.6	-2.9	-2.6
HFCO	3.4	7.8	10.7	4.3
HOF	-3.4	8.7	7.6	2.0
LiF	2.4	-0.8	-1.5	-0.1
LiH	-2.2	-9.3	-6.0	-2.1
N_2	2.8	1.5	5.6	0.9
N_2O	0.3	7.1	6.5	3.1
NH_3	-4.0	-3.1	-1.6	-2.4
O_3	235.4	58.6	59.4	136.6
OCS	-3.9	8.0	9.1	0.7
OF_2	-4.4	26.5	25.0	7.3
PN	14.3	15.8	23.5	10.8
SO_2	17.3	15.9	21.6	13.6
MAE*	4.7	7.9	8.6	3.7
ME*	0.1	5.7	7.3	2.4
STD*	6.5	8.8	8.8	4.9

* Statistics for the mean absolute error (MAE), mean error (ME) and the standard deviation (STD) exclude O_3.

Table S15: Deviations of the magnetizabilities computed with TURBOMOLE and GIMIC for the CAMh-B3LYP, CAM-QTP-00, and CAM-QTP-01 functionals in the aug-cc-pCVQZ basis set from CCSD(T) data from ref. S2 in units of 10^{-30}J/T^2.

Molecule	CAMh-B3LYP	CAM-QTP-00	CAM-QTP-01
AlF	-2.4	-0.0	-2.7
C_2H_4	7.1	0.9	4.0
C_3H_4	12.7	6.5	7.0
CH_2O	11.8	2.9	12.1
CH_3F	2.1	-1.0	-0.9
CH_4	-1.7	-0.6	-5.8
CO	1.6	4.3	0.1
FCCH	0.4	-3.2	-1.9
FCN	1.2	-1.8	-1.2
H_2C_2O	0.3	-3.1	-3.5
H_2O	-2.1	1.1	-2.9
H_2S	-0.7	1.1	-3.4
H_4C_2O	5.7	-3.0	-0.4
HCN	0.6	-2.1	-2.1
HCP	4.4	-1.7	3.0
HF	-2.4	0.9	-2.7
HFCO	5.0	1.9	2.7
HOF	2.9	-3.3	-0.2
LiF	0.1	2.9	-0.3
LiH	-2.6	2.3	-1.4
N_2	1.6	2.9	-0.4
N_2O	3.9	0.9	1.4
NH_3	-1.7	0.5	-3.6
O_3	128.6	251.6	161.7
OCS	2.0	-3.9	-2.3
OF_2	9.4	-7.9	2.2
PN	12.3	10.6	7.9
SO_2	15.5	15.7	11.2
MAE*	4.2	3.2	3.2
ME*	3.2	0.9	0.6
STD*	5.2	4.7	4.5

* Statistics for the mean absolute error (MAE), mean error (ME) and the standard deviation (STD) exclude O_3.

Table S16: Deviations of the magnetizabilities computed with TURBOMOLE and GIMIC for the CAM-QTP-02, CHACHIYO, HF, KT1, KT2, and KT3 functionals in the aug-cc-pCVQZ basis set from CCSD(T) data from ref. S2 in units of 10^{-30}J/T^2.

Molecule	CAM-QTP-02	CHACHIYO	HF	KT1	KT2	KT3
AlF	-2.9	2.3	-4.7	-3.9	2.1	0.3
C_2H_4	2.6	16.6	-9.2	7.0	10.4	13.2
C_3H_4	5.6	20.2	0.8	17.4	21.6	25.7
CH_2O	10.9	18.1	-12.0	10.6	9.4	9.5
CH_3F	-1.7	4.9	-2.2	5.9	8.3	10.4
CH_4	-6.4	1.4	3.3	-3.8	0.9	5.1
CO	0.2	6.9	5.0	-4.5	0.4	3.4
FCCH	-2.9	5.3	-10.6	-3.4	1.4	4.6
FCN	-2.1	6.6	-8.0	-2.4	2.4	4.9
H_2C_2O	-4.7	4.3	-8.7	-4.2	1.8	7.0
H_2O	-2.7	-0.8	3.9	-3.7	0.1	1.3
H_2S	-4.0	1.7	2.5	-7.0	-0.6	4.3
H_4C_2O	-2.6	13.0	-9.7	8.2	13.9	18.7
HCN	-3.0	10.1	-8.3	-3.0	1.3	4.7
HCP	1.6	17.3	-18.8	-0.8	4.9	8.9
HF	-2.4	-2.0	3.7	-3.4	-0.4	-0.1
HFCO	1.7	12.5	-4.3	3.9	8.2	9.7
HOF	-1.7	8.2	-9.2	4.0	7.8	10.5
LiF	0.0	-0.6	4.8	-3.6	-0.6	1.7
LiH	-0.8	-4.5	1.9	-11.9	-9.9	-10.8
N_2	-0.7	8.2	2.3	-4.6	0.2	4.0
N_2O	0.5	7.5	-3.7	4.5	8.6	11.1
NH_3	-3.8	0.7	2.9	-3.6	0.7	3.3
O_3	182.3	62.1	457.4	10.4	17.1	27.7
OCS	-3.8	11.0	-13.4	2.0	8.5	12.5
OF_2	-1.3	25.0	-24.7	15.4	20.7	23.8
PN	7.3	28.3	4.0	6.1	11.2	17.1
SO_2	10.6	25.4	314.3	9.7	17.2	21.7
MAE*	3.3	9.8	18.4	5.9	6.4	9.2
ME*	-0.2	9.2	7.5	1.1	5.6	8.4
STD*	4.4	8.9	61.8	7.1	7.2	8.1

* Statistics for the mean absolute error (MAE), mean error (ME) and the standard deviation (STD) exclude O_3.

Table S17: Deviations of the magnetizabilities computed with Turbomole and Gimic for the LDA, M06, M06-2X, M06-L, M08-HX, and M08-SO functionals in the aug-cc-pCVQZ basis set from CCSD(T) data from ref. S2 in units of 10^{-30}J/T^2.

Molecule	LDA	M06	M06-2X	M06-L	M08-HX	M08-SO
AlF	-1.3	7.4	1.6	11.8	-2.7	-1.3
C_2H_4	14.5	13.2	15.7	18.5	14.4	11.3
C_3H_4	14.5	13.6	17.5	17.4	16.2	15.2
CH_2O	31.5	22.9	33.3	3.8	33.3	38.0
CH_3F	0.3	2.3	-1.5	4.6	-3.8	-3.3
CH_4	-12.5	0.6	-3.0	7.5	-5.7	-6.1
CO	2.9	17.2	15.6	14.2	7.8	14.3
FCCH	3.0	7.1	1.9	7.6	-0.8	0.6
FCN	4.7	11.3	5.4	10.1	1.7	4.8
H_2C_2O	-3.8	6.6	5.8	5.1	0.8	4.6
H_2O	-5.8	2.0	-0.5	4.9	-0.7	-1.6
H_2S	-10.9	3.4	-2.6	8.0	-2.0	-4.0
H_4C_2O	5.4	5.8	-5.1	14.1	-8.2	-8.3
HCN	6.7	19.4	11.4	19.2	6.6	9.4
HCP	15.3	27.8	16.4	29.9	9.5	12.3
HF	-4.7	1.3	-0.2	2.6	-0.6	-1.1
HFCO	10.3	15.1	14.3	14.8	11.5	13.7
HOF	6.4	7.1	0.3	5.4	-1.0	0.1
LiF	-0.8	5.1	2.1	3.7	2.3	2.5
LiH	-8.8	-3.1	-0.9	-0.5	-1.9	-2.4
N_2	4.1	24.0	16.1	18.7	9.5	15.5
N_2O	4.8	12.8	6.3	10.1	3.4	7.4
NH_3	-7.8	2.1	-1.6	7.3	-1.7	-3.1
O_3	73.7	291.9	371.4	34.7	226.7	525.9
OCS	7.5	13.4	5.8	14.7	0.6	3.9
OF_2	26.8	18.6	4.2	12.8	0.1	5.5
PN	23.6	58.8	48.3	41.2	24.5	48.3
SO_2	19.2	38.0	36.9	28.9	26.6	42.5
MAE*	9.6	13.3	10.1	12.5	7.3	10.4
ME*	5.4	13.1	9.0	12.4	5.2	8.1
STD*	11.4	13.2	13.1	9.4	10.3	14.3

* Statistics for the mean absolute error (MAE), mean error (ME) and the standard deviation (STD) exclude O_3.

Table S18: Deviations of the magnetizabilities computed with TURBOMOLE and GIMIC for the M11, M11-L, MN12-L, MN12-SX, and MN15 functionals in the aug-cc-pCVQZ basis set from CCSD(T) data from ref. S2 in units of 10^{-30}J/T^2.

Molecule	M11	M11-L	MN12-L	MN12-SX	MN15
AlF	3.0	-8.6	-12.6	-8.9	-5.7
C_2H_4	14.3	11.5	5.2	7.2	15.2
C_3H_4	12.3	17.6	7.8	11.8	18.9
CH_2O	38.4	-8.0	-17.6	-1.0	36.4
CH_3F	-5.0	7.9	1.9	0.2	0.8
CH_4	-6.8	4.9	1.3	-0.8	-2.5
CO	10.3	6.1	-1.9	1.7	15.2
FCCH	1.6	-3.3	-5.0	-3.6	4.4
FCN	4.3	1.9	-1.7	-0.9	8.9
H_2C_2O	-0.4	-7.5	-10.7	-4.9	7.6
H_2O	-1.3	7.3	4.6	1.7	-0.0
H_2S	-4.0	4.5	1.0	0.3	0.3
H_4C_2O	-10.0	16.7	9.7	2.7	3.7
HCN	9.1	7.9	-1.7	0.2	12.0
HCP	20.9	3.8	-8.7	-2.1	11.3
HF	-1.5	6.6	2.9	1.2	-0.4
HFCO	13.5	6.9	1.8	3.9	18.1
HOF	0.6	6.8	0.6	-2.0	5.3
LiF	1.5	7.2	4.2	4.7	0.0
LiH	0.8	-22.8	-17.8	-13.3	-4.3
N_2	11.5	11.5	0.9	3.9	18.3
N_2O	4.6	8.4	3.0	3.0	10.7
NH_3	-2.4	6.0	4.0	0.8	-0.7
O_3	362.9	-36.6	-75.9	9.6	449.5
OCS	1.2	4.2	-3.2	-1.9	9.8
OF_2	5.8	4.8	-2.7	-6.3	16.8
PN	42.0	14.7	-22.0	-2.8	36.6
SO_2	40.9	23.4	2.0	11.0	45.4
MAE*	9.9	8.9	5.8	3.8	11.4
ME*	7.6	5.2	-2.0	0.2	10.4
STD*	13.8	9.3	8.0	5.3	12.8

* Statistics for the mean absolute error (MAE), mean error (ME) and the standard deviation (STD) exclude O_3.

Table S19: Deviations of the magnetizabilities computed with TURBOMOLE and GIMIC for the MN15-L, MVS, N12, N12-SX, PBE, and PBE0 functionals in the aug-cc-pCVQZ basis set from CCSD(T) data from ref. S2 in units of 10^{-30}J/T^2.

Molecule	MN15-L	MVS	N12	N12-SX	PBE	PBE0
AlF	-15.7	10.2	0.4	-0.2	-2.4	0.2
C_2H_4	2.4	25.8	14.5	11.8	14.8	10.3
C_3H_4	8.5	19.3	22.6	15.1	19.4	13.8
CH_2O	-15.0	3.9	14.5	14.1	22.5	14.6
CH_3F	1.6	6.6	7.9	1.9	4.5	1.3
CH_4	-1.9	1.8	1.3	0.4	-3.4	-1.5
CO	-5.8	11.5	4.3	8.4	3.9	6.6
FCCH	-8.6	4.7	8.2	5.4	4.0	1.7
FCN	-6.7	4.3	9.2	7.4	5.0	3.4
H_2C_2O	-14.2	-4.9	7.3	4.3	2.1	0.3
H_2O	-0.7	2.8	0.8	1.4	-3.4	-0.1
H_2S	-3.9	-0.9	3.7	1.2	-3.5	-1.1
H_4C_2O	5.1	15.4	18.6	7.0	11.3	3.7
HCN	-5.2	15.5	6.5	7.8	7.4	5.8
HCP	-15.7	25.1	15.6	12.7	13.5	10.1
HF	-0.5	1.6	0.2	0.4	-3.7	-0.7
HFCO	-2.4	9.8	12.4	11.6	10.4	8.5
HOF	-4.1	8.7	12.8	5.4	8.0	2.6
LiF	-1.8	1.0	3.8	2.2	-0.7	1.4
LiH	-10.7	1.7	-11.9	-1.7	-8.0	-1.9
N_2	-3.5	13.3	5.4	9.7	5.4	7.0
N_2O	-0.9	6.2	12.6	9.2	7.3	4.9
NH_3	-1.6	2.9	1.4	1.7	-2.8	-0.1
O_3	-57.9	15.1	63.8	171.4	61.9	136.1
OCS	-6.6	8.1	15.7	7.4	9.3	4.5
OF_2	-7.9	18.5	29.5	13.4	25.5	8.8
PN	-22.3	30.5	22.3	25.0	23.9	23.2
SO_2	-4.1	24.5	30.9	30.1	20.5	22.8
MAE*	6.6	10.4	10.9	8.0	9.1	6.0
ME*	-5.3	9.9	10.0	7.9	7.1	5.6
STD*	6.9	9.2	9.6	7.5	9.4	6.8

* Statistics for the mean absolute error (MAE), mean error (ME) and the standard deviation (STD) exclude O_3.

Table S20: Deviations of the magnetizabilities computed with TURBOMOLE and GIMIC for the QTP-17, revB3LYP, revM06, and revM06-L functionals in the aug-cc-pCVQZ basis set from CCSD(T) data from ref. S2 in units of 10^{-30}J/T^2.

Molecule	QTP-17	revB3LYP	revM06	revM06-L
AlF	-2.9	-2.4	4.8	8.6
C_2H_4	7.1	8.6	16.0	19.0
C_3H_4	14.8	15.3	16.3	17.7
CH_2O	10.6	13.6	23.0	-6.6
CH_3F	3.0	2.9	0.5	5.2
CH_4	-0.0	-1.4	-1.5	4.9
CO	2.4	2.7	12.3	3.1
FCCH	0.5	1.2	3.4	3.8
FCN	1.6	2.3	5.8	3.5
H_2C_2O	1.1	1.0	2.8	-3.8
H_2O	-1.5	-2.0	0.4	4.3
H_2S	-0.2	-1.2	-1.2	8.2
H_4C_2O	7.0	7.4	1.8	17.9
HCN	1.0	2.2	10.5	7.1
HCP	2.8	5.3	16.7	14.4
HF	-1.8	-2.2	0.2	2.0
HFCO	5.5	6.5	12.8	8.3
HOF	3.2	4.0	2.7	3.6
LiF	0.9	0.7	1.5	0.4
LiH	-3.7	-4.1	-0.6	3.3
N_2	2.5	3.0	13.5	4.7
N_2O	4.7	5.0	5.9	4.5
NH_3	-0.9	-1.7	-0.3	5.8
O_3	129.9	118.0	274.2	-27.6
OCS	2.9	4.1	6.0	7.0
OF_2	9.9	12.9	9.0	6.1
PN	14.0	15.9	38.4	3.0
SO_2	17.2	17.9	33.4	12.5
MAE*	4.6	5.4	8.9	7.0
ME*	3.8	4.3	8.7	6.2
STD*	5.4	6.1	10.3	6.0

* Statistics for the mean absolute error (MAE), mean error (ME) and the standard deviation (STD) exclude O_3.

Table S21: Deviations of the magnetizabilities computed with Turbomole and Gimic for the revM11, revTPSS, revTPSSh, rSCAN, and SCAN functionals in the aug-cc-pCVQZ basis set from CCSD(T) data from ref. S2 in units of 10^{-30}J/T^2.

Molecule	revM11	revTPSS	revTPSSh	rSCAN	SCAN
AlF	1.0	1.3	1.8	-0.5	2.5
C_2H_4	12.6	13.6	11.9	13.3	12.6
C_3H_4	11.5	21.2	18.7	15.5	16.5
CH_2O	27.7	4.2	2.8	5.5	1.0
CH_3F	-1.1	6.8	5.4	3.9	5.3
CH_4	-3.2	8.1	7.6	-1.6	2.5
CO	7.6	4.6	5.4	2.8	2.6
FCCH	3.8	4.9	3.8	2.4	3.3
FCN	4.6	4.8	4.1	3.4	4.2
H_2C_2O	5.2	7.6	6.2	-3.4	-2.1
H_2O	-3.0	-0.5	0.5	0.1	0.9
H_2S	-1.1	6.6	6.4	-2.4	1.2
H_4C_2O	-3.0	14.5	11.3	10.6	15.4
HCN	7.7	7.0	6.3	5.4	6.5
HCP	19.7	11.7	10.3	10.3	11.1
HF	-3.5	-2.1	-1.1	-0.4	-0.1
HFCO	9.1	9.2	8.3	8.4	7.9
HOF	1.6	5.1	3.2	5.3	4.5
LiF	-1.4	-0.7	0.2	-0.4	-0.1
LiH	3.8	-1.3	-0.0	-3.7	-3.2
N_2	9.7	6.2	6.5	3.8	5.4
N_2O	5.1	7.8	6.8	5.5	6.5
NH_3	-2.7	2.8	3.2	-0.0	1.6
O_3	324.0	21.0	46.4	17.2	21.9
OCS	3.3	10.5	8.3	6.1	6.4
OF_2	8.1	15.9	10.3	14.7	12.8
PN	34.2	20.1	19.6	13.4	15.9
SO_2	27.0	22.6	22.8	16.8	17.9
MAE*	8.2	8.2	7.1	5.9	6.3
ME*	6.8	7.9	7.1	5.0	5.9
STD*	10.0	6.7	5.9	6.1	6.0

* Statistics for the mean absolute error (MAE), mean error (ME) and the standard deviation (STD) exclude O_3.

Table S22: Deviations of the magnetizabilities computed with TURBOMOLE and GIMIC for the TASK, TPSS, TPSSh, ωB97, ωB97M-V, and ωB97X functionals in the aug-cc-pCVQZ basis set from CCSD(T) data from ref. S2 in units of 10^{-30}J/T^2.

Molecule	TASK	TPSS	TPSSh	ωB97	ωB97M-V	ωB97X
AlF	11.2	0.6	1.2	-2.5	-5.6	-1.0
C_2H_4	20.2	13.5	11.8	7.5	5.1	8.1
C_3H_4	18.1	20.4	18.0	7.4	9.0	9.3
CH_2O	-5.5	7.4	5.7	8.5	11.6	11.0
CH_3F	6.4	6.2	4.8	0.4	0.7	0.8
CH_4	6.2	5.3	5.1	-1.8	-4.1	-1.0
CO	5.0	4.8	5.6	1.3	-1.0	3.7
FCCH	2.5	4.9	3.8	-0.5	-3.0	0.4
FCN	2.2	5.2	4.5	-0.3	-1.8	1.5
H_2C_2O	-7.4	6.0	4.8	-3.1	-3.3	-0.7
H_2O	3.9	-0.6	0.4	-0.8	-2.2	-0.0
H_2S	0.8	4.5	4.6	0.9	-3.1	0.8
H_4C_2O	16.1	13.9	10.7	1.3	1.3	2.7
HCN	9.7	7.4	6.7	2.3	-2.6	3.2
HCP	14.6	12.3	10.9	10.9	1.8	9.0
HF	2.7	-2.0	-0.9	-2.0	-2.3	-0.8
HFCO	8.6	9.4	8.6	3.3	3.2	6.2
HOF	4.3	6.1	4.1	0.9	0.1	1.9
LiF	0.4	0.2	0.9	-2.4	-0.8	-1.7
LiH	6.8	-2.3	-0.8	0.8	-6.7	-2.1
N_2	7.5	6.4	6.8	2.8	-1.9	4.6
N_2O	3.4	8.3	7.3	1.4	1.6	3.1
NH_3	4.2	1.9	2.5	0.0	-2.5	0.4
O_3	21.4	29.5	55.4	115.4	103.9	139.0
OCS	3.3	10.5	8.3	-1.5	-2.2	0.9
OF_2	10.3	18.2	12.3	3.7	1.7	6.0
PN	19.1	20.9	20.5	13.6	7.5	16.0
SO_2	22.8	22.4	22.7	13.5	10.5	17.8
MAE*	8.3	8.2	7.2	3.5	3.6	4.2
ME*	7.3	7.8	7.1	2.4	0.4	3.7
STD*	7.4	6.8	6.0	4.8	4.8	5.2

* Statistics for the mean absolute error (MAE), mean error (ME) and the standard deviation (STD) exclude O_3.

Table S23: Deviations of the magnetizabilities computed with TURBOMOLE and GIMIC for the ωB97X-D, and ωB97X-V functionals in the aug-cc-pCVQZ basis set from CCSD(T) data from ref. S2 in units of 10^{-30}J/T^2.

Molecule	ωB97X-D	ωB97X-V
AlF	1.4	-2.0
C_2H_4	10.1	6.7
C_3H_4	12.0	9.0
CH_2O	12.7	8.8
CH_3F	1.6	0.8
CH_4	0.8	-0.8
CO	7.1	2.1
FCCH	1.8	-0.8
FCN	3.8	-0.1
H_2C_2O	1.8	-1.4
H_2O	1.3	-0.9
H_2S	2.2	0.9
H_4C_2O	5.1	2.4
HCN	5.6	1.1
HCP	10.1	6.3
HF	0.5	-1.7
HFCO	9.3	4.1
HOF	3.4	0.7
LiF	-0.1	-0.9
LiH	-4.3	-0.2
N_2	7.5	2.3
N_2O	4.8	2.3
NH_3	1.6	-0.2
O_3	145.7	129.7
OCS	4.5	-0.6
OF_2	9.1	3.3
PN	21.7	12.2
SO_2	23.7	14.5
MAE*	6.2	3.2
ME*	5.9	2.5
STD*	6.3	4.4

* Statistics for the mean absolute error (MAE), mean error (ME) and the standard deviation (STD) exclude O_3.

Table S24: Comparison of the magnetizabilities in 10^{-30}J/T^2 calculated with TURBOMOLE (TM) /GIMIC employing the resolution of the identity approximation, and PySCF employing exact integrals at the BP86/aug-cc-pCVQZ and B3LYP/aug-cc-pCVQZ levels of theory. The PySCF data is in full agreement with that from GAUSSIAN.

Molecule	BP86			B3LYP		
	TM/GIMIC	PySCF	difference	TM/GIMIC	PySCF	difference
AlF	-394.3	-394.4	0.2	-396.5	-396.6	0.1
C_2H_4	-331.0	-330.9	-0.0	-336.7	-336.7	-0.0
C_3H_4	-460.1	-460.1	-0.0	-463.1	-463.0	-0.0
CH_2O	-108.1	-108.1	0.0	-114.9	-114.9	0.0
CH_3F	-311.4	-311.4	0.0	-312.4	-312.4	0.0
CH_4	-318.5	-318.6	0.1	-317.0	-317.1	0.1
CO	-205.2	-205.2	0.0	-206.6	-206.6	0.0
FCCH	-437.9	-437.9	-0.0	-440.1	-440.0	-0.0
FCN	-365.0	-365.0	0.0	-367.4	-367.4	-0.0
H_2C_2O	-422.0	-422.1	0.1	-422.1	-422.2	0.1
H_2O	-237.5	-237.5	0.1	-236.7	-236.7	0.0
H_2S	-456.6	-456.7	0.2	-455.1	-455.3	0.2
H_4C_2O	-523.5	-523.5	0.0	-526.9	-526.9	-0.0
HCN	-264.4	-264.4	0.0	-269.4	-269.4	0.0
HCP	-479.2	-479.3	0.0	-487.4	-487.4	-0.0
HF	-179.3	-179.3	0.0	-178.4	-178.4	0.0
HFCO	-296.5	-296.5	0.0	-300.5	-300.5	0.0
HOF	-227.8	-227.7	-0.0	-231.1	-231.1	-0.0
LiF	-197.0	-197.1	0.1	-194.7	-194.8	0.1
LiH	-133.2	-133.2	-0.0	-130.8	-130.7	-0.0
N_2	-199.6	-199.5	-0.1	-202.0	-201.9	-0.1
N_2O	-332.6	-332.7	0.1	-333.8	-334.0	0.1
NH_3	-291.9	-292.0	0.1	-291.2	-291.3	0.1
O_3	180.9	181.2	-0.3	238.7	239.0	-0.3
OCS	-575.0	-575.1	0.1	-579.6	-579.7	0.1
OF_2	-222.1	-221.8	-0.2	-234.1	-233.9	-0.2
PN	-284.7	-284.3	-0.4	-292.2	-291.8	-0.4
SO_2	-292.7	-292.3	-0.5	-296.1	-295.6	-0.4

References

(S1) Valiev, R. R.; Fliegl, H.; Sundholm, D. Closed-shell paramagnetic porphyrinoids. *Chem. Commun.* **2017**, *53*, 9866–9869.

(S2) Lutnæs, O. B.; Teale, A. M.; Helgaker, T.; Tozer, D. J.; Ruud, K.; Gauss, J. Benchmarking density-functional-theory calculations of rotational g tensors and magnetizabilities using accurate coupled-cluster calculations. *J. Chem. Phys.* **2009**, *131*, 144104.
